# Tetrametallic Au@Ag-Pd-Pt Nanozyme with Surface-Exposed Active Sites for Enhanced Catalytic Activity

**DOI:** 10.3390/nano15231833

**Published:** 2025-12-04

**Authors:** Vasily G. Panferov, Nadezhda A. Byzova, Konstantin B. Shumaev, Anatoly V. Zherdev, Boris B. Dzantiev

**Affiliations:** A.N. Bach Institute of Biochemistry, Research Center of Biotechnology of the Russian Academy of Sciences, Leninsky Prospect 33, Moscow 119071, Russianbyzova@inbi.ras.ru (N.A.B.); tomorov@mail.ru (K.B.S.); zherdev@inbi.ras.ru (A.V.Z.)

**Keywords:** nanozymes, galvanic replacement, catalysis, peroxidase-mimicking activity, synthesis of nanoparticles, polymetallic nanoparticles

## Abstract

Metal nanoparticles (NPs) with enzyme-mimicking activities, known as nanozymes, are being actively explored for biomedical and analytical applications. Enhancing their catalytic activity and metal utilization efficiency is crucial for advancing these technologies. Here, we report an aqueous-phase, room-temperature synthesis of tetra-metallic Au@Ag-Pd-Pt NPs that exhibit superior peroxidase-like activity compared to their mono-, bi-, and trimetallic counterparts. The synthesis involves a sequential, seed-mediated approach comprising the formation of Au NP seeds, the overgrowth of a Ag shell, and the galvanic replacement of Ag with Pd and Pt ions. We systematically investigated the effects of the Au core diameter (15, 40, 55 nm), Ag precursor concentration (50–400 µM), and the Pd-to-Pt ratio on the optical and catalytic properties. By changing the particle composition, we were able to tune the absorbance maximum from 520 nm to 650 nm while maintaining high extinction coefficients (10^9^–10^10^ M^−1^cm^−1^) comparable to that of the initial Au nanoparticles. Mapping of chemical element distributions in the nanoscale range confirmed a core–shell–shell architecture with surface-enriched Pd and Pt. This structure ensures the surface-exposed localization of catalytically active atoms, yielding a more than 10-fold improvement in specific peroxidase-like activity while utilizing up to two orders of magnitude less Pt and Pd than bimetallic particles. The synthesized NPs thus combine high catalytic activity with tunable optical properties, making them promising multifunctional labels for biosensing.

## 1. Introduction

Nanoparticles that mimic the catalytic activity of enzymes, termed nanozymes, are widely utilized in biomedical and analytical fields [1,2,3,4]. They are often considered more stable and cost-effective alternatives to natural enzymes and possess unique properties not found in their biological counterparts, such as localized surface plasmon resonance, magnetism, and tunable catalytic activities [5]. While these properties are advantageous, the catalytic activity, defined by parameters like turnover number and specific activity, remains the most critical characteristic [6]. However, the catalytic activity of nanozymes is often lower than that of natural enzymes, making the development of strategies to enhance it a primary focus in nanozymology.

A common strategy for increasing nanozyme activity is to maximize the number of accessible active sites [7]. In conventional spherical nanoparticles larger than 20 nm, over 90% of atoms are buried within the bulk and do not participate in catalysis. In contrast, enzymes achieve extraordinary efficiency by concentrating metal ions in exposed active sites [8]. Inspired by this, the field of single-atom catalysts has emerged [9]. Although these systems offer high atom utilization, their complex synthesis and low operational stability often limit their practical application. For conventional nanozymes, maximizing active sites can be achieved by altering the nanoparticle morphology (e.g., creating nanorods or 2D sheets) or by depositing a thin layer of catalytically active material onto a carrier particle [1]. For instance, Xia’s group demonstrated that ultra-slow precursor addition can control surface deposition and diffusion [10], leading to significant activity enhancements in core@shell nanostructures, though this approach has been limited to two-component systems such as Pd-Ir [11], Au-Pt [12], Au-Ag [13] and Pd-Pt [14].

Another promising approach is to modulate the chemical composition. Multi-metallic nanoparticles often exhibit synergistic effects that boost catalytic activity beyond that of monometallic nanozymes [15]. The enhancement of catalytic activity in multi-metallic nanozymes, often termed synergy, can arise from several electronic and geometric effects [16,17]. Alloying can lead to strain effects and ligand (electronic) effects that optimize the binding energy of reaction intermediates, a key principle in heterogeneous catalysis [18]. The combination of multiple metals into a single catalyst, forming multi-metallic nanozymes, has been reported to enhance catalytic efficiency in various reactions, including the oxygen reduction reaction [19], oxygen evolution reaction [20], hydrogen evolution reaction [21], and CO oxidation [22]. The development of nanozymes that integrate multiple elements is an actively progressing research direction [23]. Several mechanisms have been proposed to explain this improved activity, such as increased surface area, mixed cation valences, a greater number of active sites, and alterations in electronic and phase structure [24]. High-entropy alloys represent a parallel development in this field [25]. The combination of multiple elements in such systems induces a lattice distortion effect, which can create sites with higher energy states conducive to catalysis, and a synergistic “cocktail” effect arising from the interaction of the various components. However, for both high-entropy alloys and multi-metallic nanozymes, the discovery of these synergistic effects and the associated improvement in catalytic activity mainly relies on an empirical trial and error approach. Therefore, the development of universal, rational synthesis strategies for multi-metallic nanozymes remains a goal of high importance.

Platinum group and noble metals (such as Au, Ag, Pd, Pt, Ir) are of particular interest due to their high intrinsic catalytic activity, chemical stability, and versatility for functionalization [26,27]. However, their high cost restricts widespread application and sharply underscores the necessity of maximizing metal utilization efficiency in catalysis [28]. Recently, nanozymes with more than three metallic elements, inspired by high-entropy alloys, have been reported [29]. However, their synthesis typically relies on complex, multistep procedures involving high temperatures, restricting their preparation to well-equipped, specialized laboratories. Moreover, the challenge of integrating multiple metals with high atom utilization in a single nanostructure remains largely unaddressed. Conventional overgrowth and co-reduction methods for synthesizing multi-metallic NPs inherently bury the most catalytically valuable metals within the core, resulting in poor atom utilization efficiency [30].

This work combines the principles of surface-exposed active sites and multi-metallic synergy through the design of tetra-metallic NPs, synthesized in aqueous phase, featuring a Au core and a Ag shell with Pd and Pt atoms. Pd and Pt atoms were introduced by galvanic replacement, so that they were located in surface-exposed areas and not buried inside particles as typically observed for conventional overgrowth methods. We demonstrate that the size, optical properties, and catalytic performance of these NPs can be tuned by varying precursor concentrations, and we observed a systematic increase in peroxidase-like activity through the series of bimetallic (Au@Ag), trimetallic (Au@Ag-Pd) and tetra-metallic (Au@Ag-Pd-Pt) NPs.

## 2. Materials and Methods

### 2.1. Materials

HAuCl_4_, AgNO_3_, Na_2_PtCl_6_, trisodium citrate, L-ascorbic acid sodium salt, polyvinylpyrrolidone (PVP, MW 30,000), hydrogen peroxide (H_2_O_2_, 30%), and 3,3′,5,5′-tetramethylbenzidine (TMB) were purchased from Sigma-Aldrich (St. Louis, MO, USA). Na_2_PdCl_4_ was obtained from the Tokyo Chemical Industry (Tokyo, Japan). All solutions were prepared using MilliQ water (Millipore Corporation, Burlington, MA, USA).

### 2.2. Synthesis of Au NPs

Au NPs were synthesized via the citrate reduction of HAuCl_4_ according to the method developed by Frens [31]. Briefly, 95 mL of deionized water was combined with 1 mL of a 1% (*w*/*v*) HAuCl_4_ solution in a round-bottom flask equipped with a reflux condenser and heated to boiling. Under vigorous stirring, 4 mL of a 1% (*w*/*v*) trisodium citrate solution was rapidly injected. The solution was boiled for 30 min. The final size of the Au NPs was controlled by varying the volume of the 1% trisodium citrate solution added to a fixed 100 mL total reaction volume (4 mL for 15 nm, 1.5 mL for 40 nm, and 1 mL for 55 nm particles). The molar concentrations of the resulting Au NPs are reported in Table S1.

### 2.3. Synthesis of Au@Ag NPs

Au@Ag core–shell NPs were prepared by the ascorbate-mediated reduction of AgNO_3_ onto the Au NP seeds [32]. First, the as-synthesized Au NPs were purified by centrifugation (17,000× *g*, 15 min) and pellets were redispersed in an equal volume of water. All subsequent steps were performed on ice using pre-cooled (4 °C) solutions to ensure uniform shell growth and prevent aggregation. For a typical synthesis, 4 mL of Au NPs was mixed with 125 µL of sodium ascorbate (50 mM) and stirred rapidly for 1 min. Then, 375 µL of a freshly prepared AgNO_3_ solution (concentrated 12-fold relative to the desired final concentration) was added manually at a controlled rate of 100 µL/min. The reaction was allowed to proceed for 30 min on ice with continuous stirring.

### 2.4. Synthesis of Au@Ag-Pd-Pt

The Au@Ag NPs were incubated with 0.1% (*w*/*v*) PVP for 30 min. The PVP-coated NPs were then centrifuged and redispersed in an equal volume of water. For the galvanic replacement reaction, 600 µL of the purified Au@Ag NPs was mixed with 100 µL of freshly prepared solutions of Na_2_PdCl_4_ and Na_2_PtCl_6_. The concentrations of the Pd and Pt precursors were calculated based on the stoichiometry of the galvanic replacement reactions, where the deposition of one Pd atom requires the oxidation of two Ag atoms, and the deposition of one Pt atom requires the oxidation of four Ag atoms.

### 2.5. Characterization of NPs

The size and morphology of the nanoparticles were characterized using transmission electron microscopy (TEM) on a Jeol CX-100 (Jeol, Tokyo, Japan) and a Tecnai Osiris FEI microscopes (Hillsboro, OR, USA). Elemental composition and mapping were performed using energy-dispersive X-ray spectroscopy on the Tecnai Osiris FEI. UV-Vis spectra were recorded using either a Biochrom Libra S80 (Cambridge, UK) cuvette spectrophotometer or a CLARIOstar microplate reader (Offenburg, Germany). Kinetic measurements of peroxidase-like activity and the calculation of Michaelis–Menten constants and specific activities were conducted according to the procedures detailed in our previous work [32]. The peroxidase-like kinetics were characterized using the Au_55nm_Ag_200µM_Pd_50%_Pt_50%_ nanoparticles (140 µg/mL). The nanoparticles were incubated with a solution containing 0.5 mM TMB and varying concentrations of H_2_O_2_ (0–3000 mM) in 20 mM sodium acetate buffer (pH 4.0). The kinetics of TMB oxidation were monitored by the increase in absorbance at 650 nm for 2 min. The initial reaction rate was determined from the linear slope within the first 40–60 s. Specific peroxidase-like activity was determined according to the method described by Jiang et al. [33]. Serial dilutions of NPs were incubated with a substrate solution containing 0.5 mM TMB and 1500 mM H_2_O_2_ in 20 mM sodium acetate buffer (pH 4.0). The initial rates were calculated and converted into the rate of TMB oxidation (µM/s) using the molar extinction coefficient for oxidized TMB (ε_650_ = 39,000 M^−1^ cm^−1^). Formation of hydroxyl radicals was monitored by electron paramagnetic resonance (EPR). 5,5-Dimethyl-1-pyrroline N-oxide (DMPO) was used as a spin trap. EPR spectra were measured at ambient temperature (25 °C) using an X-band EPR spectrometer E-109E (Varian, USA). The instrument settings were as follows: modulation frequency, 100 kHz; time constant, 0.032; microwave power, 10 mW; microwave frequency, 9.15 GHz; and modulation amplitudes: 0.1 mT for DMPO spin adducts. For the assay, Au_60nm_Ag_400µM_Pd_20%_Pt_40%_ nanoparticles (1 µg/mL) were mixed with freshly prepared 55 mM DMPO and 150 mM H_2_O_2_, followed by a 5 min incubation. A Fenton reaction induced by 0.5 mM FeSO_4_ served as a positive control.

## 3. Results

### 3.1. General Strategy

**Figure 1:**
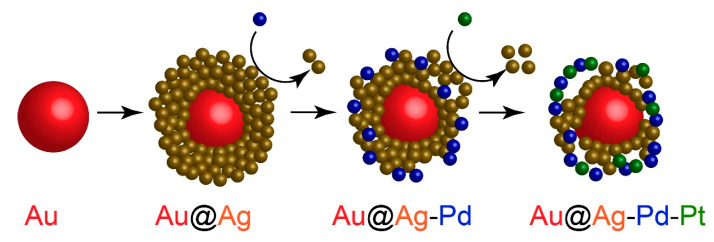
Schematics of NP synthesis.

The synthesis of tetra-metallic nanoparticles involved sequential stages: (1) synthesis of spherical Au nanoparticle seeds, (2) formation of a Ag shell over the Au seeds to form Au@Ag core–shell nanoparticles, and (3) partial galvanic replacement of the Ag shell with Pd and Pt by introducing PdCl_4_^2−^ and PtCl_6_^2−^ ions (Figure 1). We utilized three different diameters of Au seeds (15, 40 and 55 nm) to investigate the effect of core size. This variation not only changes the total surface area available for Ag deposition but also, for a fixed mass concentration of gold, inversely affects the number of seed particles (Table S1). Consequently, these factors collectively influence the ultimate thickness of the Ag shell and the loading of Pd and Pt atoms per individual nanoparticle.

The silver shell was formed by reducing silver nitrate with sodium ascorbate in the presence of the Au NP seeds. To ensure the formation of a uniform and smooth shell, the reaction was carried out at a low temperature with a controlled, slow addition of the silver nitrate precursor at a rate of 100 µL/min. Using this method, a total of 12 distinct Au@Ag core–shell samples were synthesized. This library was generated by systematically varying two parameters: the core size (15, 40, and 55 nm) and the concentration of silver nitrate precursor (50, 100, 200, and 400 µM). The samples were designated accordingly (Au_15nm_Ag_50µM_, Au_15nm_Ag_100µM_, Au_15nm_Ag_200µM_, and Au_15nm_Ag_400µM_, and similarly for Au_40nm_ and Au_55nm_).

The silver shell functions as a sacrificial template for the galvanic deposition of Pd and Pt. Galvanic replacement is a thermodynamically driven redox process that proceeds at room temperature without requiring external reducing agents. The driving force is the difference in reduction potentials between the sacrificial metal and the depositing metal ion, where the depositing metal must have a higher standard reduction potential [34]. Silver, with its relatively low reduction potential (Ag^+^/Ag = 0.80), is a common sacrificial template for ions like PdCl_4_^2−^ (Pd^2+^/Pd = 0.91) [35] and PtCl_6_^2−^ (Pt^4+^/Pt = 1.18) [34]. While powerful, the application of galvanic replacement has been largely confined to bimetallic systems [36]. In this work, we extended it to create tri- and tetra-metallic nanostructures. The process was performed in two sequential steps: First, we performed the addition of PdCl_4_^2−^ ions to Au@Ag particles to form trimetallic Au@Ag-Pd NPs, followed by the addition of PtCl_6_^2−^ ions to yield the final tetra-metallic Au@Ag-Pd-Pt NPs. We confirmed that the sequence of ion addition (PdCl_4_^2−^ before PtCl_6_^2−^ or vice versa) had no significant effect on morphology and catalytic activity of the final product (Figure S1), although an excessive concentration of PtCl_6_^2−^ was avoided to prevent the potential replacement of previously deposited Pd.

The stoichiometry of the reaction dictates the final morphology. The reduction of one Pd atom (from PdCl_4_^2−^) requires the oxidation of two Ag atoms, while the reduction of one Pt atom (from PtCl_6_^2−^) requires four Ag atoms. Because the number of oxidized Ag atoms exceeds the number of deposited Pd or Pt atoms, the process leads to the formation of porous, cage-like structures with internal voids [37]. This mechanism ensures that the deposited Pd and Pt atoms are directly surface-exposed, rather than buried, making galvanic replacement an ideal strategy for maximizing atom utilization efficiency [32]. To prevent the precipitation of nanoparticles by AgCl, which forms from the Ag^+^ ions released during the reaction, the Au@Ag nanoparticles were stabilized with a coating of polyvinylpyrrolidone (PVP) prior to galvanic replacement. Au@Ag particles capped with PVP were centrifuged and redispersed in H_2_O to remove free PVP. The excess of free PVP could complex with the Pt and Pd precursors [38], affecting their reactivity. Potentially, alternative non-halide complexes of Pd and Pt could be used to avoid the precipitation of poorly soluble AgCl. However, most current protocols utilize chloride precursors and instead employ various methods to remove the formed AgCl, such as treatment with ammonium hydroxide [39], HCl/NaCl washing [40,41] or heating [42].

Based on the stoichiometry of the galvanic replacement reactions, we denote our NPs to reflect the percentage of the maximum possible Pd and Pt deposition, as determined by the available Ag. For example, a sample named Au_55nm_Ag_200µM_Pd_50%_Pt_50%_ indicates that 50% of the sacrificial Ag was replaced by Pd deposition and 50% by Pt. To establish general trends, we studied the effect of multiple variables, including Au core size, Ag shell thickness, and Pd/Pt ratio, on the morphology, optical properties, and catalytic activity of the resulting NPs. Given that the total number of synthesized NPs exceeded 60, key composition–property relationships were established using a selected set of preparations that effectively represented the entire parameter space. This approach allowed us to generalize our conclusions and establish robust trends linking composition to morphology and, ultimately, to functional properties.

### 3.2. Characterization of the Morphology of NPs

**Figure 2:**
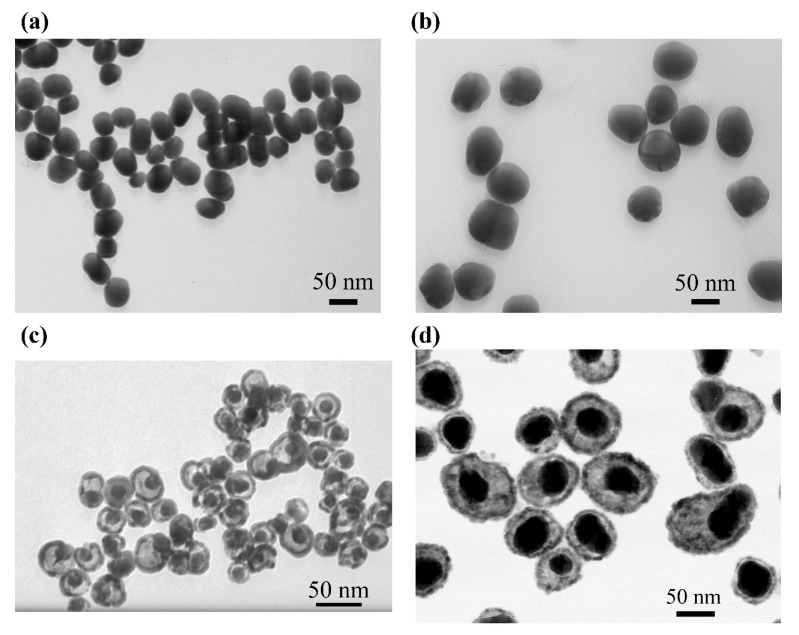
TEM of NPs. (**a**) Au_55nm_ NPs. (**b**) Au_55nm_Ag_400µM_. (**c**) Au_15nm_Ag_100µM_Pd_50_. (**d**) Au_40nm_Ag_400µM_Pd_25_Pt_40_.

The spherical Au nanoparticles synthesized as seeds were characterized by transmission electron microscopy (TEM). As shown in Figure 2a, the particles were monodisperse and largely spherical with no observable aggregation, confirming their suitability for subsequent seed-mediated growth. The mean diameters were determined to be 15.9 ± 1.2 nm, 40.6 ± 5.5 nm, and 53.3 ± 4.0 nm. These are referred to throughout the text as Au_15nm_, Au_40nm_, and Au_55nm_, respectively.

TEM analysis of the Au@Ag nanoparticles confirmed high monodispersity and a notable absence of aggregates (Figure 2b and Figure S2). Maintaining a low temperature during synthesis was critical, as elevated temperatures often lead to the “soldering” of Au cores, particularly at high silver nitrate concentrations. The optimized conditions employed here successfully prevented such aggregation, cross-linking, and homogeneous nucleation of free-standing Ag NPs. The final size of the Au@Ag NPs exhibited a clear correlation with both the concentration of silver nitrate and the diameter of the initial Au seeds. For instance, using 15 nm Au seeds, the mean diameter increased from 18.0 ± 1.8 nm (at 100 µM AgNO_3_) to 25.1 ± 2.3 nm (at 400 µM AgNO_3_), confirming tunable and controlled overgrowth. Due to differences in the total surface area and population density of the seed particles, the resulting Ag shell thickness varied for Au_15nm_, Au_40nm_, and Au_55nm_ seeds even at identical silver nitrate concentrations.

Both the trimetallic (Au@Ag-Pd) and tetra-metallic (Au@Ag-Pd-Pt) NPs displayed the characteristic porous and cage-like morphology indicative of galvanic replacement (Figure 2c,d). Given that their synthesis shares the same underlying mechanism, a similar morphology is expected. This structural evolution is a direct consequence of the process stoichiometry, wherein a greater number of Ag atoms are oxidized and removed than the number of Pd or Pt atoms deposited. Furthermore, the larger atomic radius of Ag and its face-centered cubic lattice constant compared to Pd and Pt contribute to the formation of these porous frameworks with regions of low electron density.

### 3.3. Characterization of Composition of NPs

**Figure 3:**
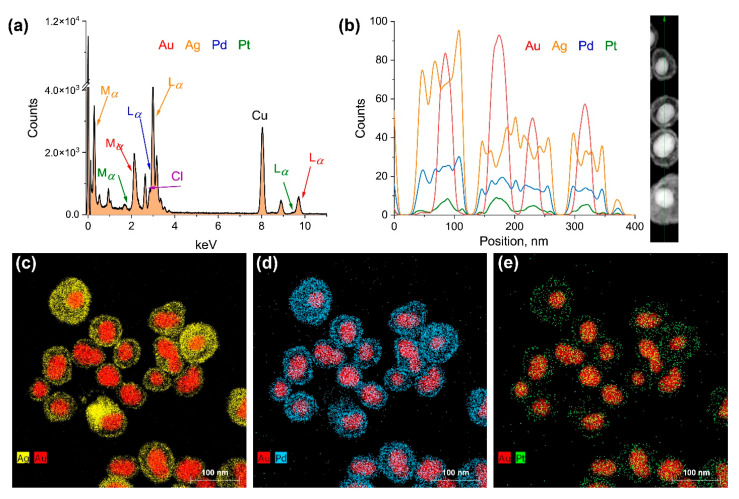
Characterization of chemical composition of Au_40nm_Ag_400µM_Pd_40%_Pt_40%_. (**a**) EDS spectrum confirming the presence of Au, Ag, Pd, and Pt. (**b**) EDS line profile across a cluster of nanoparticles, showing the distribution of elements. (**c**–**e**) TEM-EDS elemental maps of a single representative nanoparticle: (**c**) Au (red) and Ag (yellow), (**d**) Au (red) and Pd (blue), and (**e**) Au (red) and Pt (green).

While TEM effectively visualized the morphological changes, it cannot confirm the co-deposition of Pd and Pt. To verify the successful formation of tetra-metallic NPs, energy-dispersive X-ray spectroscopy (EDS) was performed. The EDS spectrum (Figure 3a) confirmed the presence of all four metallic elements (Au, Ag, Pd, Pt), with the signals for Pd and Pt being relatively lower, as anticipated given their surface-limited deposition. The detection of chlorine is consistent with the precipitation of AgCl from Ag^+^ ions released during galvanic replacement. To probe the elemental distribution, EDS line scanning and elemental mapping were employed. The line profile (Figure 3b) is characteristic of a core–shell structure, showing a Ag shell surrounding the Au core. The profiles for Pd and Pt were distributed across the entire particle, consistent with their deposition throughout the porous cage. This was confirmed by TEM-EDS elemental mapping (Figure 3c–e), which clearly visualized the localization of all four elements within a single nanoparticle. The maps show the Au core surrounded by a Ag shell, with both Pd and Pt elements scattered across the edges and surface of the structure, aligning perfectly with the expected model (Figure 1).

The final composition of the tetra-metallic nanozymes is governed by the distinct stoichiometry of the galvanic replacement reactions. The deposition of a single Pd atom requires the oxidation of two Ag atoms, whereas the deposition of a single Pt atom requires the oxidation of four Ag atoms. Consequently, for an identical nominal percentage of precursor (e.g., 40% for both Pd and Pt in Au_40nm_Ag_400µM_Pd_40%_Pt_40%_, Figure 3), the resulting atomic concentration of Pd in the final nanoparticle is twice that of Pt. This is clearly demonstrated by the higher signal intensity for Pd in the compositional analysis (Figure 3d,e).

The galvanic replacement reaction occurs exclusively at the particle–solvent interface, localizing the deposited materials at surface-exposed sites. Although the initially deposited atoms can theoretically undergo interdiffusion (e.g., via the Kirkendall effect) to form alloyed structures, the interdiffusion rate of Pd is higher than that of Pt. To test this, we estimated the relative concentration of Pd at the particle edges versus the center. If our hypothesis of surface enrichment is valid, a higher Pd concentration should be found near the edges (i.e., the original Ag shell). If interdiffusion rate is sufficient, alloy articles with no enrichment of Pd at specific regions will be detected.

Quantitative EDS analysis of the atomic composition (Figure 3d) confirmed this. The relative fraction of Pd at the particle center was 12.2% ± 1.2% (with a total signal of 762 ± 30 counts/s), while the concentration near the edges was significantly higher, at 36.6% ± 6.0% (total signal 98 ± 7 counts/s). Therefore, the results from both chemical mapping (Figure 3c–e) and EDS confirm that Pd atoms remain predominantly enriched at the edges and surface. This is consistent with the expected mechanism of a surface-limited galvanic replacement process.

### 3.4. Characterization of the Optical Properties of NPs

The optical properties of nanozymes, particularly in the visible region, are critically important for their use as colorimetric labels. In addition, modifications of optical properties are a sensitive indicator of changes in chemical composition and morphology of NPs [43]. The initial Au NPs serve as excellent colorimetric labels due to their high molar extinction coefficient (ε). Therefore, we systematically investigated how the sequential formation of the Ag shell and the deposition of Pd and Pt atoms affect both the position of the localized surface plasmon resonance peak (λ_max_) and the corresponding ε-values. As the absence of homogeneous nucleation was confirmed, the molar concentration of particles can be accurately estimated by the theoretical calculations of Au seed concentration (Table S1).

**Figure 4:**
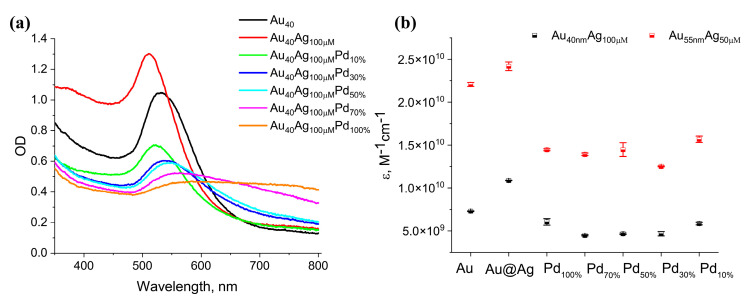
The effect of Pd loading on the optical properties of the nanoparticles. (**a**) UV-Vis spectra of NPs. (**b**) Molar extinction coefficients at the λ_max_ wavelength.

The overgrowth of the Ag shell onto the Au seeds resulted in a blue-shift of the localized λ_max_, accompanied by a substantial increase in the optical density (Figure 4a). This enhancement ε-values makes Au@Ag core–shell nanoparticles even more efficient colorimetric labels than the initial Au NPs. Subsequent galvanic replacement of the sacrificial Ag shell induced a pronounced red-shift of the λ_max_ to wavelengths longer than those of the original Au NPs, coupled with a strong decrease in the optical density (Figure 4a), yielding violet-blue colloidal solutions. The deposition of Pt onto the trimetallic Au@Ag-Pd NPs did not induce further significant spectral changes. Consequently, the final λ_max_ positions of the tri- and tetra-metallic nanoparticles were similar and were predominantly determined by the size and shape of the initial Au seeds. This indicates that tailoring the Au core architecture is an effective strategy for controlling the optical properties of the resulting multi-metallic nanostructures.

The utility of nanoparticles as a colorimetric label is primarily governed by their ε-values, and maximizing this parameter is key to developing highly sensitive bioassays [44]. Our results confirm that the bimetallic Au@Ag NPs possess the highest ε values among all samples studied. Galvanic replacement led to a reduction in ε (Figure 4b). However, galvanic replacement also resulted in a broadening of the absorption peak and increased optical density at longer wavelengths (>600 nm), as seen in Figure 4a. For applications relying on bare-eye readout, such as lateral flow assays, the perceived intensity of coloration, which integrates absorption across the visible spectrum, is more critical than the absorbance at a single wavelength. To evaluate this, we quantified the total area under the extinction spectrum from 350 to 800 nm (integrated intensity), as reported by Bai et al. [45]. These results (Figure S3) revealed that the trimetallic NPs maintained integrated extinction values comparable to the initial Au NPs, with trends similar to those observed for the molar extinction coefficient.

In summary, although the galvanic replacement process reduces the peak ε-value, the resulting tri- and tetra-metallic NPs retain remarkable optical properties, with integrated extinction performance comparable to Au NPs, which are often considered the “gold standard” for colorimetric labels. Therefore, these multifunctional nanoparticles are promising not only as catalytic nanozymes but also as intrinsic colorimetric reporters in biosensing applications.

### 3.5. Characterization of the Catalytic Properties of NPs

The synthesized nanoparticles exhibited intrinsic peroxidase-like activity, catalyzing the oxidation of TMB in the presence of H_2_O_2_. It is important to note that the AgCl precipitate that formed during galvanic replacement was found to suppress catalytic activity and turnover number (*k_cat_*) (Figure S4), despite having no effect on the optical properties. For some nanoparticles (e.g., Au_40nm_Ag_100µm_Pd_100%_, Table S2), the lack of a AgCl removal step resulted in particle aggregation of particles and a consequent dramatic reduction in catalytic activity. Therefore, prior to all catalytic assays, NPs were treated with ammonium hydroxide (NH_4_OH, 10 mM) to dissolve AgCl [39], followed by multiple rounds of centrifugation to remove dissolved species and ensure the removal of the alkaline solution. EDS demonstrated a significant reduction in the peak at 2.62 keV after treatment with NH_4_OH (Figure S5A). This peak corresponds to the Kα emission line of chlorine, confirming the effective dissolution of AgCl. Because the AgCl layer was thin, no changes in nanoparticle morphology were observed (Figure S5B,C).

We confirmed that the bi-, tri-, and tetra-metallic nanoparticles exhibit peroxidase-like activity, which qualifies them as nanozymes. It is important to note that all nanozymes used in this study were coated with PVP, which partially blocks the catalytic surface, resulting in reduced catalytic activity [46]. However, synthesis without the PVP capping agent often resulted in excessive aggregation. Therefore, despite the cost of reduced activity, we used only PVP-coated particles to ensure colloidal stability. For instance, the aggregated Au_40nm_Ag_100µm_Pd_100%_ demonstrated more than 27-times lower *k_cat_* value compared to the non-aggregated particles (Table S2).

**Figure 5:**
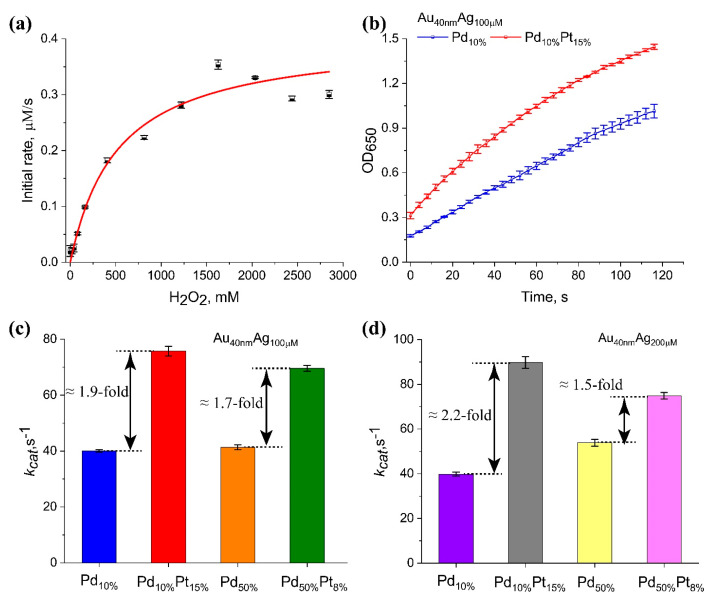
Peroxidase-like catalytic activity of NPs. (**a**) Michaelis–Menten kinetics of TMB oxidation for Au_55nm_Ag_200µM_Pd_50%_Pt_50%_ NP. (**b**) Initial reaction rates of TMB oxidation for NPs with varying Pd and Pt compositions. (**c**) *k_cat_* values for Au_40nm_Ag_100µM_ NPs with different Pd/Pt loads. (**d**) *k_cat_* values for Au_40nm_Ag_200µM_ NPs with different Pd/Pt loads.

Kinetic analysis of TMB oxidation revealed a Michaelis–Menten constant (*K_M_*) for H_2_O_2_ of 320 ± 20 µM (Figure 5a). The oxidase-like activity (TMB oxidation in the absence of H_2_O_2_) of the particles was negligible. While the Au@Ag core–shell NPs themselves possessed moderate peroxidase-like activity, we focused on the enhancement provided by the deposition of Pd and Pt. The effect was studied using Au_40nm_Ag_100µM_ and Au_40nm_Ag_200µM_. In total, eight NPs particles with various Pd and/or Pt loads (Pd_10%_, Pd_10%_Pt_15%_, Pd_50%_, Pd_50%_Pt_8%_ for each type of Au@Ag) were prepared and used for kinetic measurements. The initial rates of TMB oxidation were higher for the tetra-metallic (Au@Ag-Pd-Pt) NPs compared to their trimetallic (Au@Ag-Pd) counterparts (Figure 5b). For a more quantitative comparison, we calculated *k_cat_* for selected NPs. The calculations were based on the total molar concentration of nanoparticles (recalculated from the concentration of Au seeds), an approach that typically underestimates the absolute activity but allows for a valid comparative analysis between different nanostructures [47,48]. However, this recalculation (based on the molar concentration of nanoparticles) is highly relevant for bioanalytical applications where the analytical signal originates from nanozyme-labeled complexes, such as in a nanozyme–antibody–antigen conjugate [6]. The initial rates of TMB oxidation (Figure 5b) for 14 various tri- and tetra-metallic nanozymes were calculated from kinetic curves at saturating H_2_O_2_ concentrations (2000 mM). The values of *k_cat_* (Table S2) were determined as the ratio of the reaction rate (nM/s) to the molar concentration of particles (nM). The results (Figure 5c) demonstrate that the galvanic deposition of Pt significantly boosted the catalytic efficiency. For instance, adding 15% Pt to the Au_40nm_Ag_100µM_Pd_10%_ NP resulted in an almost two-fold increase in *k_cat_*. A similar improvement was observed for Au_40nm_Ag_100µM_Pd_50%_ even at a lower Pt load (8%). Comparable improvements in *k_cat_* (2.2-fold for Pd_10%_Pt_15%_ and 1.5-fold for Pd_50%_Pt_8%_, Figure 5d) were observed for Au_40nm_Ag_200µM_ NPs, which confirms that tetra-metallic NPs are more efficient peroxidase-like nanozymes. 

Although nanozymes catalyze typical enzyme reactions, the exact mechanisms of enzyme and nanozyme catalysis typically differ [49]. Nanozymes of various compositions often generate reactive oxygen species, which determine their analytical and biomedical applications [50]. Hydroxyl radicals are among the most commonly reported reactive oxygen species generated by nanozymes [51], and peroxidase-like oxidation of substrates is generally associated with the generation of these radicals [52]. We confirmed that Au_60nm_Ag_400µM_Pd_20%_Pt_40%_ generates hydroxyl radicals (Figure S6), which are able to oxidize TMB.

**Figure 6:**
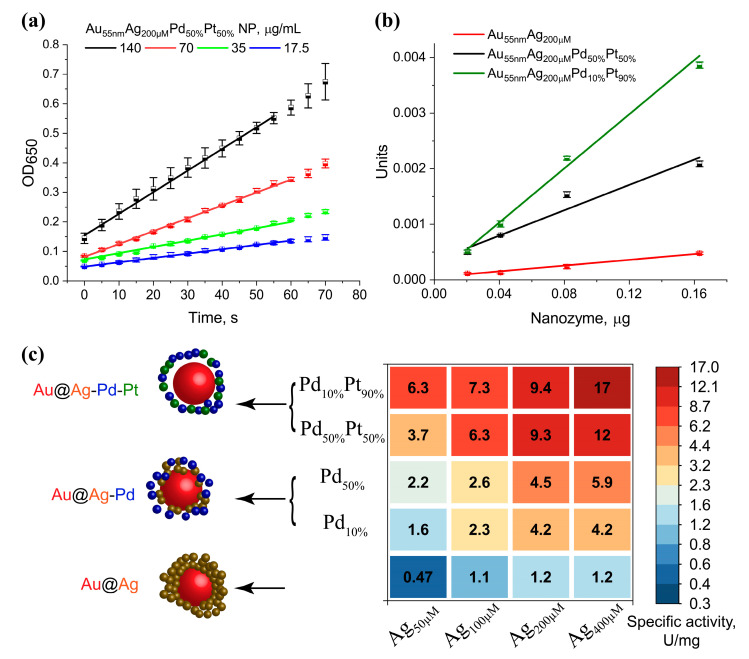
Specific peroxidase-like activity of tetra-metallic NPs. (**a**) Estimation of the initial rate of the reactions for various dilutions of NPs. (**b**) Effect of nanozyme amount on catalytic activity unit. (**c**) The values of specific activity of Au_55nm_-based NPs, showing the influence of Ag shell thickness and Pd/Pt ratio.

To establish general composition–activity relationships, we evaluated a broad panel of twenty NPs based on the Au_55nm_ core with varying Ag shell thicknesses (50–400 µM AgNO_3_) and Pd/Pt ratios. The specific activity (activity per mass of catalyst) was used as the quantitative parameter for this high-throughput screening (Figure 6a). The mass of the core–shell particles was calculated based on the mass of the Au seeds. This is a reliable approach, as no homogeneous nucleation (i.e., the formation of standalone Ag-Pd-Pt particles not grown on Au seeds) was detected. The initial rates of TMB oxidation were first determined using various mass loads of the nanozymes (Figure 6a). Nanozyme activity was quantified from the initial rates (ΔOD_650_/Δt), derived from the linear regions of the kinetic plots (R^2^ ≥ 0.98, shown as lines in Figure 6a). One unit of nanozyme activity (U) was defined as the amount of nanozyme required to oxidize 1 µM of substrate per minute under the specified conditions. Consistent with the Michaelis–Menten kinetic model, the reaction rate showed a linear relationship with the amount of catalyst. Subsequently, the initial rates were plotted against the mass of particles (Figure 6b), and the slope of this line was defined as the specific activity of the nanozyme (U/mg). The resulting data of specific activity (Figure 6c) provide clear insights into the synergistic effects of the Ag shell thickness and the Pd/Pt loading ratio on the peroxidase-mimicking activity.

Deposition of a low concentration of Pd (Pd_10%_) onto Au@Ag cores resulted in a 2–3.5-times increase in specific peroxidase activity (Figure 6c). A further increase in Pd load (Pd_50%_) provided a relatively moderate 1.1–1.5-times increased activity compared to trimetallic particles with Pt_10%_ loads, while improving the activity compared to Au@A up to 5 times. Subsequent loading of these trimetallic particles with Pt resulted in a comparable increase in specific activity, by a factor of 2–4 for Ag_50µM_Pd_10–50%_ (e.g., from 1.6 to 6.3 for Ag_50µM_ Pd_10%_, and from 2.2 to 3.7 for Ag_50µM_Pd_50%_), 2–3 for Ag_100µM_Pd_10–50%_, 2–2.5 for Ag_200µM_Pd_10–50%_, and 2–4 for Ag_400µM_Pd_10–50%_.

The fact that both sequential doping steps, first with Pd and then with Pt, yielded improvements of a similar magnitude indicates that both metals contribute significantly to the overall catalytic performance. The observed activity is therefore not primarily attributable to Pt or Pd alone, but arises from a cooperative effect. This synergy could be explained by electronic ligand effects, where the interaction between different metals at the nanoparticle surface modifies the local electronic environment, potentially optimizing the binding energy of reaction intermediates and facilitating the electron transfer processes crucial for the peroxidase-like cycle [53].

Furthermore, the activity of the tetra-metallic nanozymes aligns with previously reported values for peroxidase-mimicking nanozymes. An accurate comparison of activities is only valid for *k_cat_* and specific activity values, whereas the commonly used assessment based solely on *K_M_* and *V_max_* can be misleading [47]. Although *k_cat_* comparison is theoretically valid, it is often impractical due to the lack of a universally accepted definition for the nanozyme active center [54]. Therefore, we compared the specific peroxidase-mimicking activities (Figure 6c), which are normalized per milligram of catalyst, which is consistently used across the literature. As shown in Table S3, the synthesized nanozymes achieve activity levels comparable to those reported for other systems, but with a significantly lower concentration of precursors (typically by one to two orders of magnitude).

A key finding of this study is the exceptional metal utilization efficiency achieved by the used synthetic approach. The total concentration of Pt employed in the galvanic replacement (10–50 µM) was one to two orders of magnitude lower than the amounts typically used to form bimetallic Au@Pt or Au@Pd core–shell nanoparticles. Remarkably, despite this reduction in precious metal input, the resulting tetra-metallic Au@Ag-Pd-Pt nanoparticles exhibited a specific catalytic activity that was comparable to, and often surpassed, that of their bimetallic counterparts. This underscores the synergistic advantage of the multi-metallic, surface-exposed structure. Catalysts that minimize the consumption of precious noble metals are of great interest from both economic and environmental perspectives. The synthesis approaches utilized here are designed to minimize the use of these high-cost materials while maximizing their efficiency. Noble metals are highly stable under various chemical and physical stresses, including elevated temperatures, extreme pH, and the presence of inhibitors [55,56]. While this stability is beneficial for prolonged catalyst lifetime, it also presents a significant challenge for metal recovery. As demonstrated in our recent paper, aqua regia is one of the few reagents capable of terminating the catalytic reaction and subsequently recovering Au, Pt, and Pd in their ionic forms, making them available for reuse [57]. In future work, we will focus on a detailed evaluation of the catalytic mechanism and a more accurate quantitative analysis of the activity normalized to the mass of the catalytically active metals (Pd and Pt) to fully elucidate the metal utilization efficiency.

The tetra-metallic Au/Ag/Pd/Pt nanozymes exhibited high colloidal stability (Figure S7) and maintained their chemical composition, as verified by unchanged optical spectra (Figure S8). In contrast, as previously reported for Au@Ag nanozymes, the silver shell was oxidized by H_2_O_2_, indicated by a significant shift in the maximum wavelength and an obvious color change of the nanoparticle solution (Figure S8B). Furthermore, the tetra-metallic nanozymes withstood multiple cycles of substrate renewal and prolonged catalysis with no significant loss of the initial reaction rate, confirming their high operational stability.

Finally, we confirmed the reproducibility of the proposed synthesis procedure by characterizing four independent batches of the Au_40nm_Ag_100µM_Pd_50%_Pt_50%_ nanozyme. The hydrodynamic diameters and initial rates of TMB oxidation were highly consistent across all batches. The variation in hydrodynamic diameter was within 10% (Figure S9), confirming the high reproducibility of the synthesis. Minor differences were observed in the population of particles sized 2–20 nm, which may be attributed to residual PVP. However, the total fraction of these particles was less than 2%, further confirming synthesis robustness. More importantly, the peroxidase-mimicking activity was highly reproducible, with the initial rates varying by approximately 10% across different batches (Figure S10). This directly demonstrates the successful and consistent reproduction of the nanozyme’s catalytic function.

## 4. Conclusions

While the relationship between composition, optical properties, and catalytic activity has been explored for bimetallic [58,59] and trimetallic [32,45] noble metal nanozymes, systems with four or more metals have not yet been systematically investigated. We have developed a robust and tunable aqueous-phase strategy for synthesizing tetra-metallic Au@Ag-Pd-Pt nanoparticles. This approach provides exceptional control over the NPs’ composition, morphology, and properties. Galvanic replacement ensures that catalytically active Pd and Pt atoms are preferentially located on the nanoparticle surface, making them accessible for substrate binding. Furthermore, the multi-metallic NPs retain tunable plasmonic properties inherited from the Au core, an advantage over conventional bimetallic core@shell NPs. The combination of high catalytic activity and strong optical extinction within a single nanostructure is particularly valuable for biosensing applications. In summary, this work presents not just another new nanozyme, but a versatile synthetic platform. The principles demonstrated here can be extended to other catalytic metals (e.g., Cu, Rh, Ru, Mn) and complex compositions, opening a pathway for the synthesis of high-entropy alloy nanozymes and other functional materials.

High-activity catalysts that minimize the consumption of precious noble metals have applications extending far beyond biosensing, reaching into industrial catalysis and environmental remediation. In industrial catalysis, many processes (from fine chemical synthesis to petroleum refining) rely on catalysts containing platinum group metals [60]. The synthesis strategy demonstrated here provides a viable pathway to reduce the cost and environmental footprint of such industrial processes. In environmental remediation, these nanozymes could serve as catalysts for degrading pollutants in wastewater [61]. Furthermore, this synthetic approach aligns with the principles of green chemistry and sustainable development. The high stability and cost of noble metals also make them prime candidates for recovery and recycling.

## Data Availability

The raw data supporting the conclusions of this article will be made available by the authors on request.

## Supplementary Materials

The following supporting information can be downloaded at https://www.mdpi.com/article/10.3390/nano15231833/s1: Table S1. Concentration of synthesized Au nanoparticle seeds; Figure S1. Effect of reagent addition order during galvanic replacement on the morphology and functional properties; Figure S2. TEM images of Au@Ag NPs; Figure S3. Integrated extinction area under the UV-Vis spectrum (350–800 nm) for various nanoparticle compositions; Figure S4. The effect of AgCl dissolution with NH_4_OH on the turnover number in the peroxidase-like TMB oxidation reaction. Figure S5. Effect of NH_4_OH treatment on the morphology and composition of nanozymes; Table S2. The values of *k_cat_* for various nanozymes; Figure S6. EPR spectra for hydroxyl radical detection; Table S3. The values of specific peroxidase activity reported for various nanozymes; Figure S7. Hydrodynamic size distribution of nanozymes; Figure S8. Stability assessment via optical spectroscopy; Figure S9. Reproducibility of nanozyme synthesis; Figure S10. Reproducibility of nanozyme peroxidase-like activity. References [62,63,64,65,66,67,68,69,70,71,72,73,74,75,76,77,78,79,80,81,82,83,84,85,86,87,88,89,90,91,92,93,94,95] are cited in the supplementary materials.
