# Supplementary material for: Tetrametallic Au@Ag-Pd-Pt Nanozyme with Surface-Exposed Active Sites for Enhanced Catalytic Activity
Source: Nanomaterials (Basel). 2025 Dec 4;15(23):1833. doi: 10.3390/nano15231833 (PMC12693165; doi:10.3390/nano15231833)
Supplement: Supplementary file 1 [file nanomaterials-15-01833-s001.zip › nanomaterials-3998086-supplementary.pdf]

Supplementary Materials

Tetrametallic Au@Ag-Pd-Pt Nanozyme with Surface-Exposed Active Sites for Enhanced Catalytic Activity

Vasily G. Panferov[1], Nadezhda A. Byzova[1], Konstantin B. Shumaev[1], Anatoly V. Zherdev[1] and Boris B. Dzantiev[1] *

[1] A.N. Bach Institute of Biochemistry, Research Center of Biotechnology of the Russian Academy of Sciences, Leninsky Prospect 33, Moscow 119071, Russia
***** Correspondence: dzantiev@inbi.ras.ru

Contents

Concentrations of Au NPs preparations....2
TEM of Au@Ag NPs....4
Integrated UV-Vis intensity of NPs.....6
Effect of AgCl Removal on Catalytic Activity....7
Effect of NH_4OH treatment....8
Values of k_{cat} for nanozymes....9
Electron paramagnetic resonance study of radical generation by nanozymes....10
Comparison of specific peroxidase activity....11
Colloidal stability of nanozymes....15
Optical spectra of nanozymes....16
Reproducibility of nanozyme synthesis....18
References....20

Concentrations of Au NPs preparations

Table S1. Concentration of synthesized Au nanoparticle seeds

#	Equation	Calculation
(1) Volume of Au NP	$V = \frac{4}{3} \times \pi \times r^3$	
	For Au_{15nm}	1.77E-24 m^3
	For Au_{40nm}	3.35E-23 m^3
	For Au_{55nm}	8.716E-23 m^3
(2) Mass of Au NP	$m_{Au} = V \times \rho$ $\rho = 19.3 \frac{g}{cm^3}$	
	For Au_{15nm}	3.41E-17 g
	For Au_{40nm}	6.46E-16 g
	For Au_{55nm}	1.68E-15 g
(3) Number of Au NPs in a colloidal solution	$C_{Au\ NP} = \frac{C_{Au}}{m_{Au}}$ $C_{Au} = 0.05\ g/L$	
	For Au_{15nm}	1.47E+12 NPs per mL
	For Au_{40nm}	7.73E+10 NPs per mL
	For Au_{55nm}	2.98E+10 NPs per mL
(4) molarity of particles in a solution	$c_{AuNP} = \frac{C_{Au\ NP}}{N_A} \times 1000$	
	For Au_{15nm}	2.435 nmol/L
	For Au_{40nm}	0.128 nmol/L
	For Au_{55nm}	0.049 nmol/L

Effect of reagent order addition during galvanic replacement

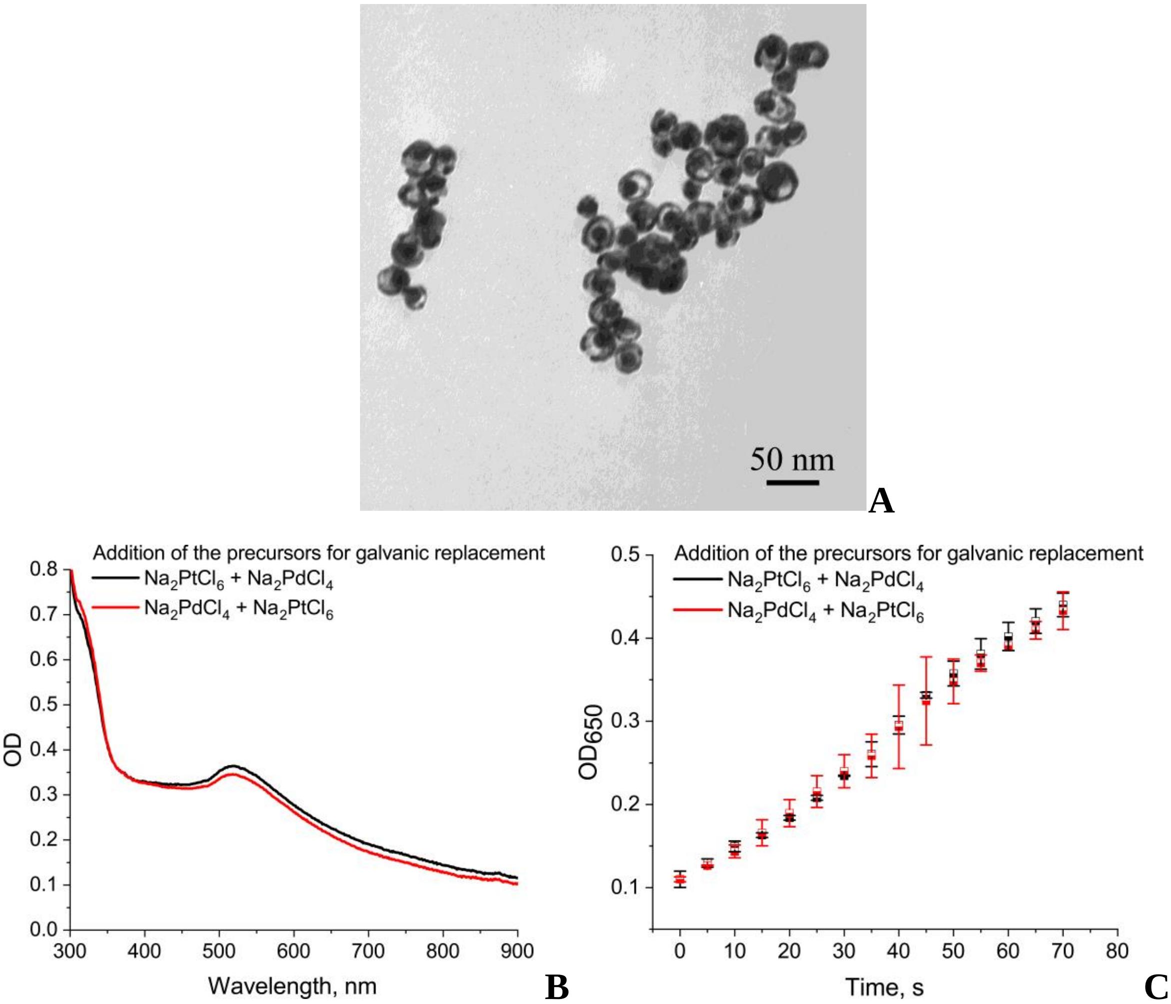

Figure S1. Effect of reagent addition order during galvanic replacement on the morphology and functional properties of $Au4_{0nm}Ag_{400\mu M}Pd_{40\%}Pt_{60\%}$ nanoparticles. (A) TEM image of the nanoparticles. (B) absorption spectra of the nanoparticles. (C) Kinetic curve of TMB oxidation catalyzed by the nanoparticles.

TEM of Au@Ag NPs

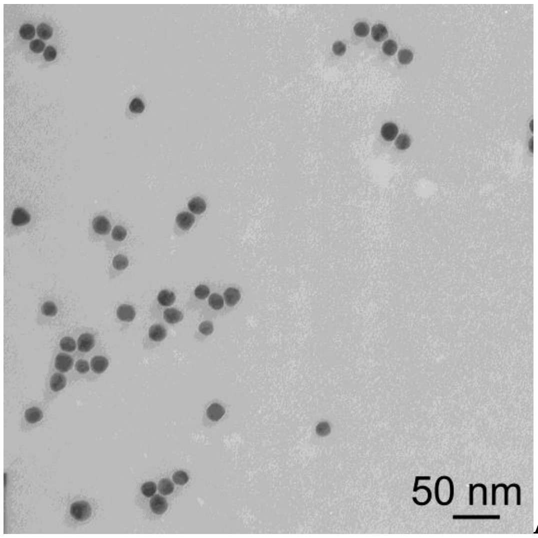

A

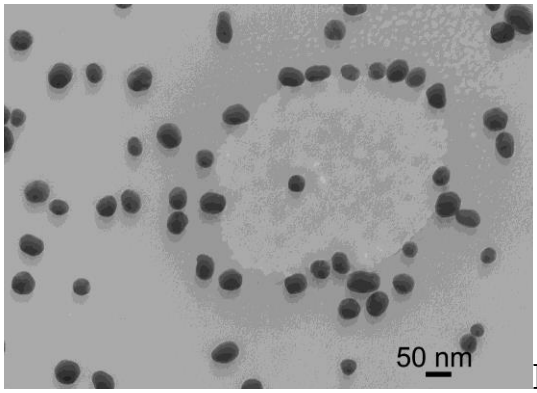

B

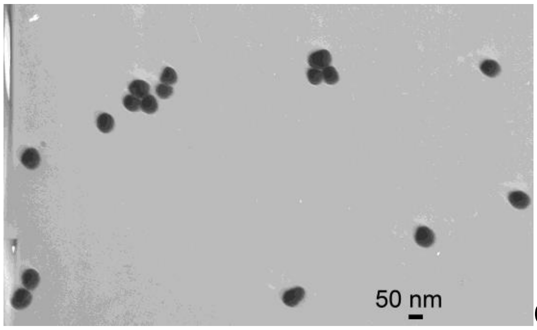

C

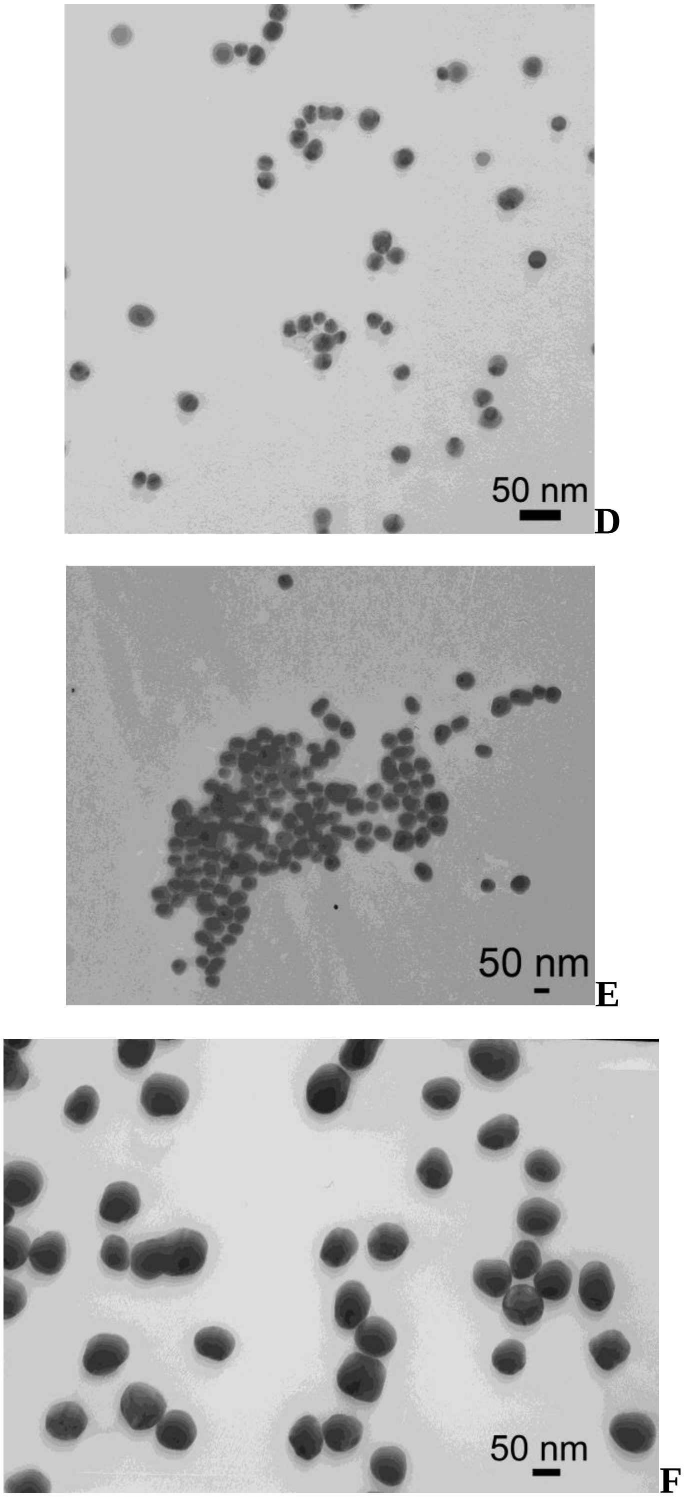

Figure S2. TEM images of Au@Ag NPs. (a) $Au_{20nm}Ag_{100\mu M}$. (b) $Au_{40nm}Ag_{100\mu M}$. (c) $Au_{55nm}Ag_{100\mu M}$. (d) $Au_{20nm}Ag_{400\mu M}$. (e) $Au_{40nm}Ag_{400\mu M}$. (f) $Au_{55nm}Ag_{400\mu M}$

Integrated UV-Vis intensity of NPs.

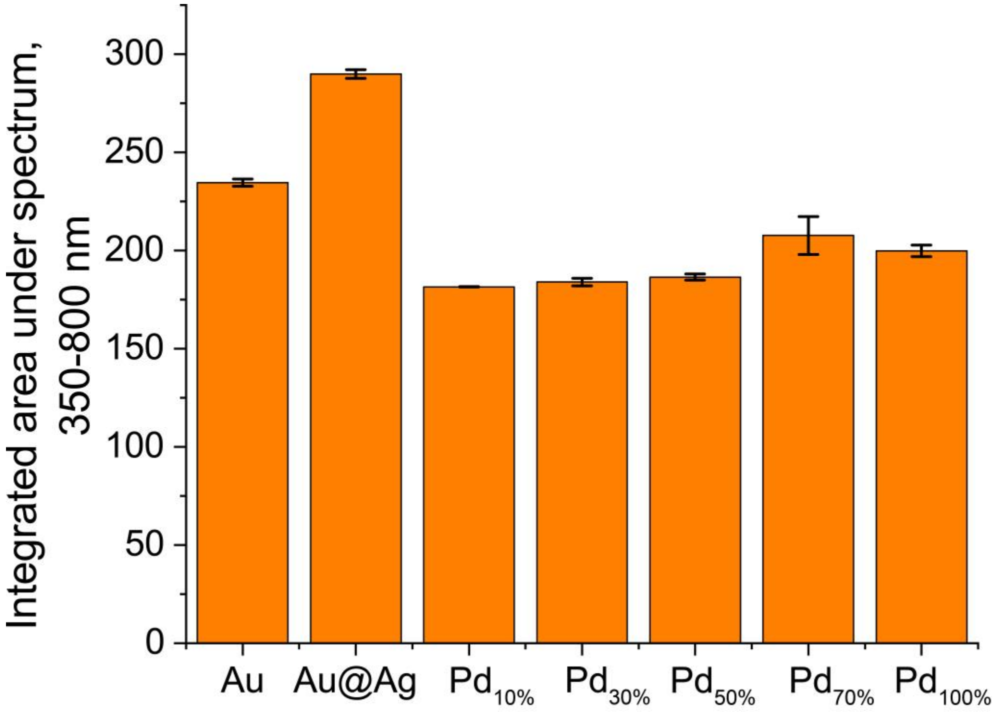

Figure S3. Integrated extinction area under the UV-Vis spectrum (350–800 nm) for various $Au_{40nm}Ag_{100\mu M}Pd_{x\%}$ compositions.

Effect of AgCl Removal on Catalytic Activity

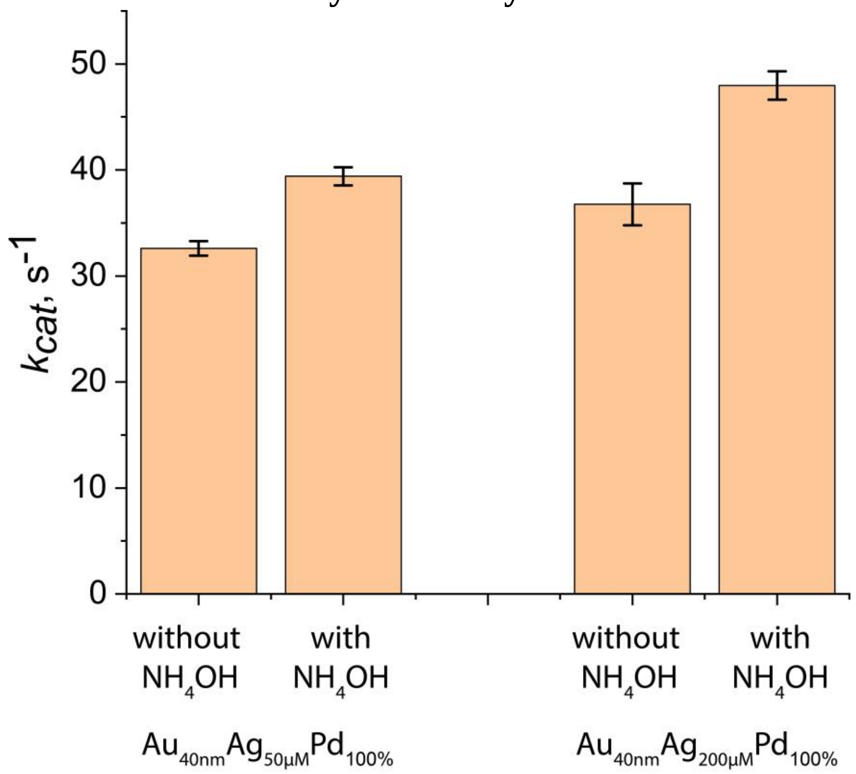

Figure S4. The effect of AgCl dissolution with NH_4OH on the turnover number in the peroxidase-like TMB oxidation reaction.

Effect of NH_4OH treatment

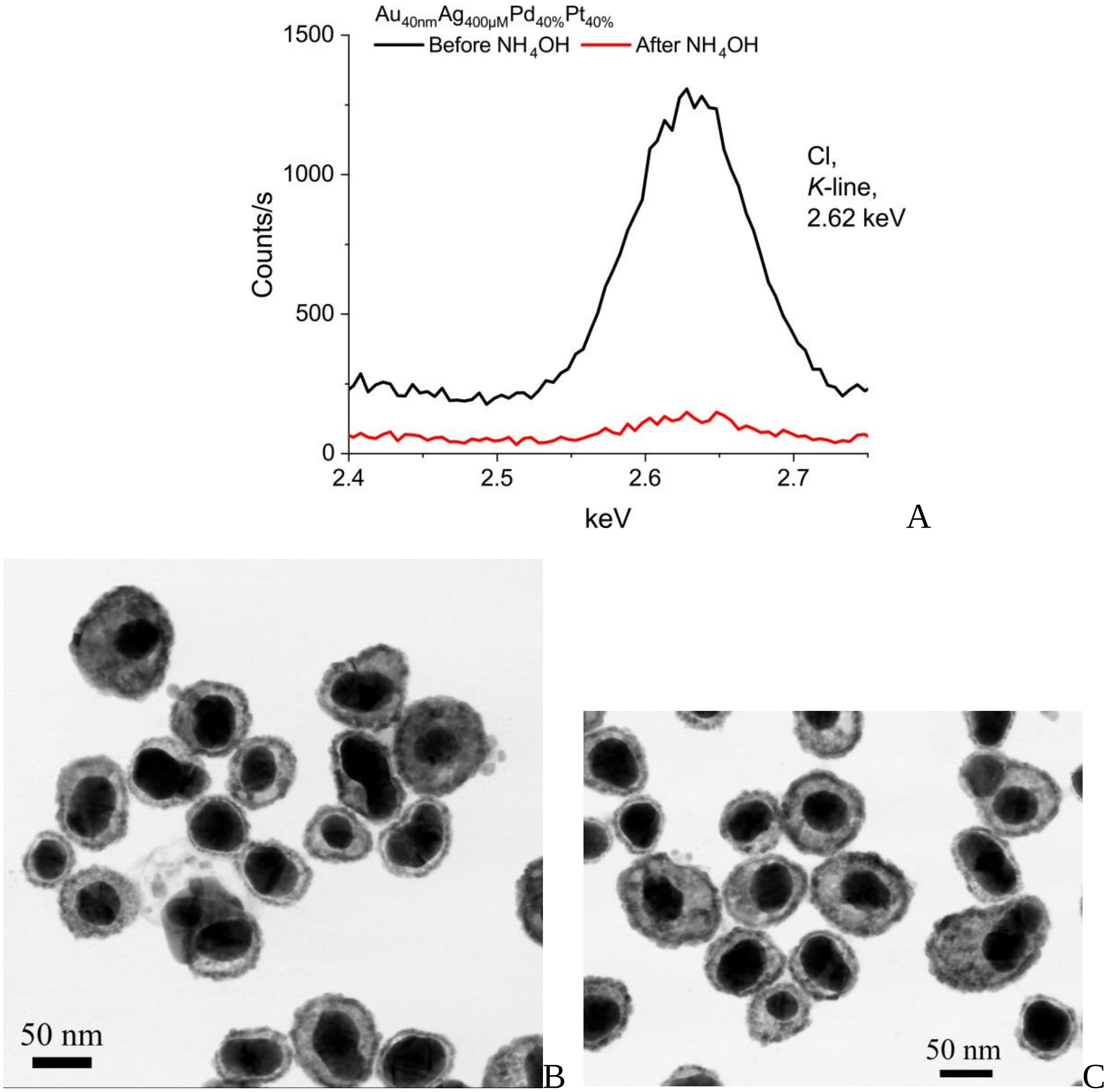

Figure S5. Effect of NH_4OH treatment on the morphology and composition of nanozymes. (A) EDS spectra of the tetrametallic nanozyme before and after treatment with 10 mM NH_4OH. (B, C) TEM images of the nanozyme (B) before and (C) after treatment with 10 mM NH_4OH.

Values of k_{cat} for nanozymes

Molarity of particles was calculated suing the initial concentrations (Table S1, (4)) and dilution factors.

Table S2. The values of k_{cat} for various nanozymes.

Composition of nanozyme	Concentration of nanozyme, nM	Rate of the reaction, nM/s	k_{cat}, s^{-1}
$Au_{40nm}Ag_{50\mu m}Pd_{100\%}$	0.091	2.97 ± 0.06	32.60 ±0.70
$Au_{40nm}Ag_{50\mu m}Pd_{100\%}$ + NH_4OH	0.091	3.59 ± 0.08	39.40 ±0.84
$Au_{40nm}Ag_{100\mu m}Pd_{10\%}$	0.091	3.65 ± 0.04	40.06 ±0.40
$Au_{40nm}Ag_{100\mu m}Pd_{50\%}$	0.091	3.77 ±0.08	41.38 ±0.90
$Au_{40nm}Ag_{100\mu m}Pd_{100\%}$ (aggregated)	0.091	0.11 ±0.01	1.25 ±0.13
$Au_{40nm}Ag_{100\mu m}Pd_{100\%}$ + NH_4OH	0.091	3.05 ±0.06	33.48 ±0.63
$Au_{40nm}Ag_{200\mu m}Pd_{10\%}$	0.091	3.63 ±0.08	39.84 ±0.86
$Au_{40nm}Ag_{200\mu m}Pd_{50\%}$	0.091	4.91 ±0.14	53.89 ±1.52
$Au_{40nm}Ag_{200\mu m}Pd_{100\%}$	0.091	3.35 ±0.18	36.77 ±1.97
$Au_{40nm}Ag_{200\mu m}Pd_{100\%}$+ NH_4OH	0.091	4.37 ±0.12	47.96 ±1.34
$Au_{40nm}Ag_{100\mu m}Pd_{10\%}Pt_{15\%}$	0.078	5.9 ±0.14	75.76 ±1.75
$Au_{40nm}Ag_{100\mu m}Pd_{50\%}Pt_{8\%}$	0.078	5.42 ±0.08	69.60 ±1.09
$Au_{40nm}Ag_{200\mu m}Pd_{10\%}Pt_{15\%}$	0.078	6.99 ±0.20	89.76 ±2.62
$Au_{40nm}Ag_{200\mu m}Pd_{50\%}Pt_{8\%}$	0.078	5.83 ±0.12	74.87 ±1.50

Electron paramagnetic resonance study of radical generation by nanozymes

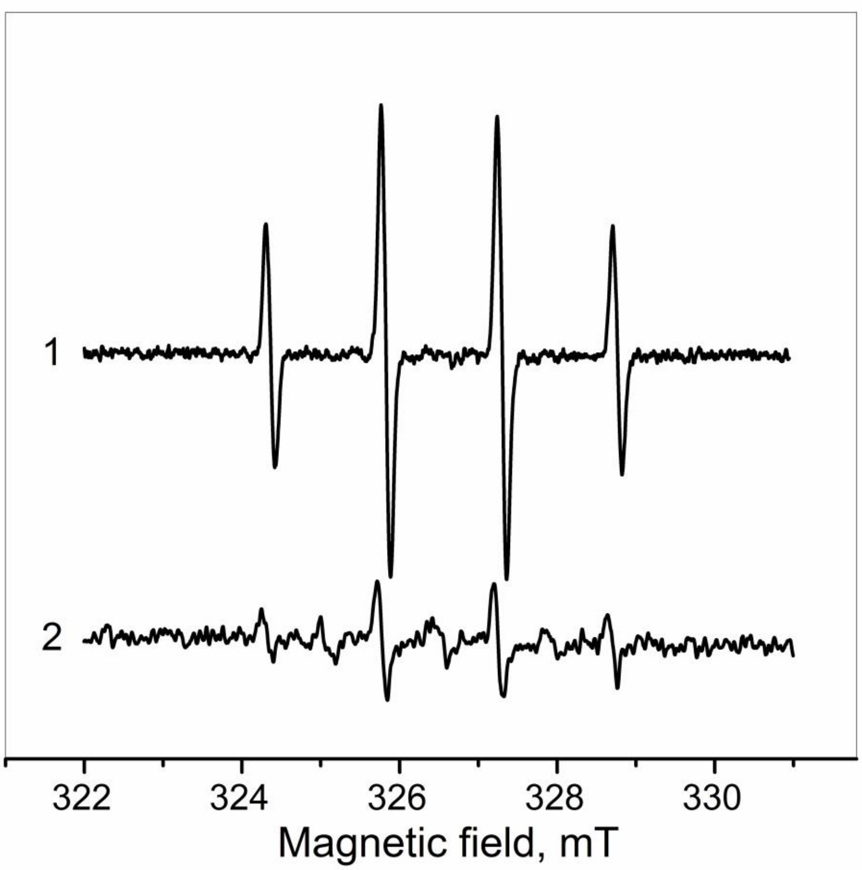

Figure S6. EPR spectra for hydroxyl radical detection. The Fenton reaction (1) served as a positive control, confirming the characteristic signal for hydroxyl radical formation. The signal from the tetrametallic nanozyme-catalyzed reaction (2) indicates the generation of the same radical species.

Comparison of specific peroxidase activity

Table S3. The values of specific peroxidase activity reported for various nanozymes. The table was adapted with modifications from Panferov et al.[1]

Nanozyme	SA	Reference
Au@Pt	3.01	[2]
Au@Pd	2.7	
Au@CeO_2	0.23	
Au@Ag	1.19	
Prussian Blue	1.10	
Au@Prussian Blue	2.16	
Cu-Prussian Blue analogue	0.48	
Pt	1.24	
Cu-PVP	0.12	
CoO	0.22	
NiO	0.025	
Au@Ag-Pt	7.43	
Au@Ag-Pd	3.25	
ZrFe-MOF-Pt	21.77	[3]
SA Fe-N_x	57.76	[4]
Au@Pt<br>with various Pt thickness	0.06<br>0.31<br>0.35<br>0.39<br>0.43<br>0.57<br>0.61<br>1.05<br>1.75<br>2.56<br>2.81<br>3.82<br>4.37<br>4.39	[5]
Pt nanoclusters-PVP<br>4 nm	 16.3	[6]

3.2 nm<br>2.1 nm	13.5<br>8.51	
Ru - polystyrene sulfonate<br>Ru - poly(acrylic acid)<br>Ru- polyvinylpyrrolidone	2820<br>521<br>1458	[7]
SA FeN_4	25.33	[8]
SA Co-N-C	6.33	
SA Zn-N-C	2.46	
SA FeN_4	6.75	[9]
Fe_3O_4	0.17	
$CoFe_2O_4$	9.90	[10]
Fe_3O_4	5	
N-doped carbon dots@Fe_3O_4	13.1	[11]
Fe_3O_4	0.215	
Co-Fe-hemin	69.915	[12]
Co-Fe	9.836	
Fe_3O_4	5.40	
FeS_2	47.98	[13]
NPS-doped carbon	7.5	[14]
NS-doped carbon	3.6	
NP-doped carbon	0.6	
N-doped carbon	0.4	
Fe_3O_4	1.79	[15]
γ-Fe_2O_3	0.45	
α-Fe_2O_3	0.03	
SA B-doped Fe-N-C	15.41	[16]
Fe-N-C	4.09	
Prussian Blue	2.46<br>2.06<br>3.13<br>1.85	[17]
Co_2FeO_4<br>55 nm<br>91 nm<br>146 nm	 5.929<br>5.511<br>5.165	[18]
Fe_3O_4<br>34 nm<br>64 nm<br>184 nm	0.404<br>0.483<br>0.292	

Au-MoS_2	12.31	[19]
MoS_2	7.44	
Au	1.28	
Various ratio Fe^{2+}/Fe^{3+}<br>oxide nanoparticles with various diameters<br>3.17 nm<br>7.82 nm<br>15.8 nm<br>21.2 nm	0.0352<br>0.1097<br>0.0876<br>0.0767	[20]
SA Co-N_2-C	1.41	[21]
SA Co-N_3-C	0.72	
SA Co-N_4-C	0.29	
Tannic acid-Ag	0.67	[22]
Ag	0.03	
Pd<br>11 nm	0.7	[23]
Pd<br>30 nm	0.91	
Pd-Ru<br>11 nm	14.82	
Pd-Ru<br>30 nm	11.21	
SA Fe-N_4	5.98	[24]
Pd	0.1497	[25]
Pd@Ir	7.44	
BSA-Os	6.12	[26]
Fe_3O_4	5.143	[27]
C nanoparticles	3.302	
Au	1.633	
Fe, N ultrathin carbon framework	36.6	[28]
Fe-N	15.3	
N, C framework	6.3	
SA Ru	7.5	[29]
Ru/C	0.38	
SA Fe S/N -doped porous carbon	79.71	[30]
SA Fe-NS-doped	24.83	
SA Fe loaded with Pt clusters	87.7	[31]
SA Fe	19.3	

Pt cluster	12.4	
SA FeN_3P	316	[32]
Fe_3O_4	9.12	
SA FeN_4	33.8	
AuPtCo	27.1	[33]
PdPtAu	81.2	[34]
Amorphous Ru	164.46	[35]
Partly crystalline Ru	77.85	
Crystalline Ru	19.62	
Fe-attapulgite-MoS_2	12.38	[36]
attapulgite	4.75	
Fe-MoS_2	3.42	
MoS_2	1.19	

Colloidal stability of nanozymes

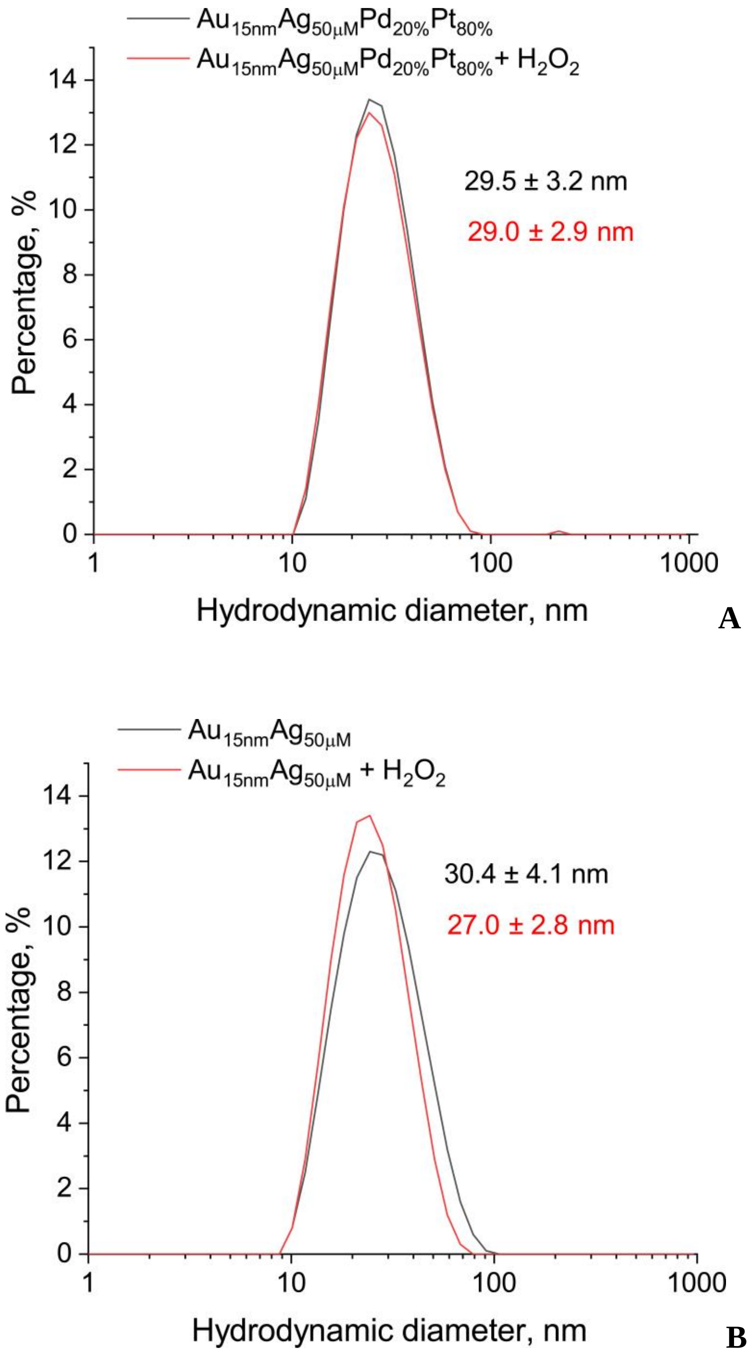

Figure S7. Hydrodynamic size distribution of nanozymes. Size distributions were measured by dynamic light scattering (DLS). (A) Tetrametallic Au/Ag/Pd/Pt nanozyme. (B) Au@Ag nanoparticles. The mean hydrodynamic diameters are indicated in the figure.

Optical spectra of nanozymes

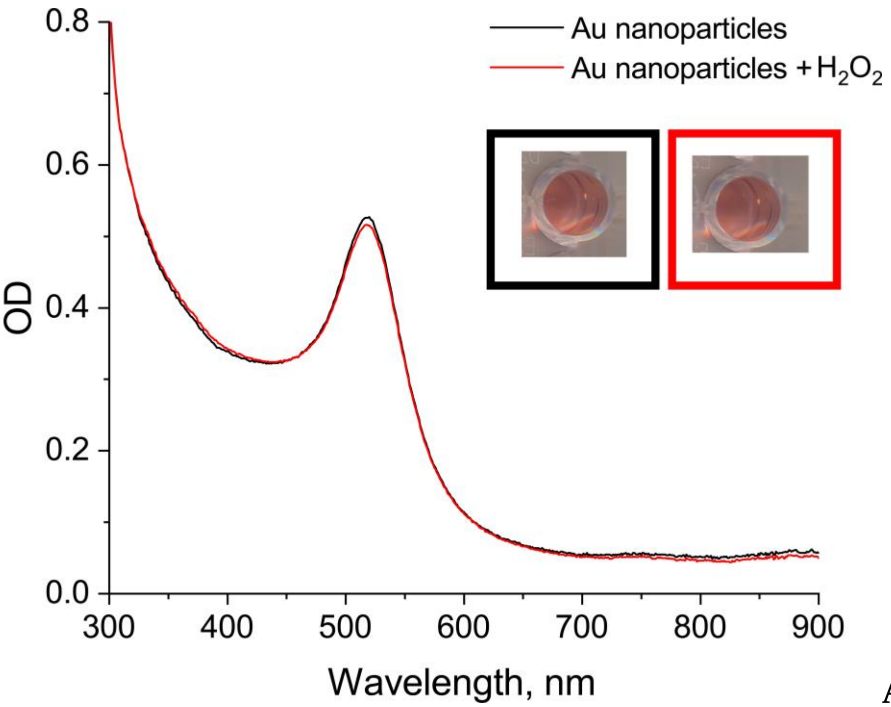

A

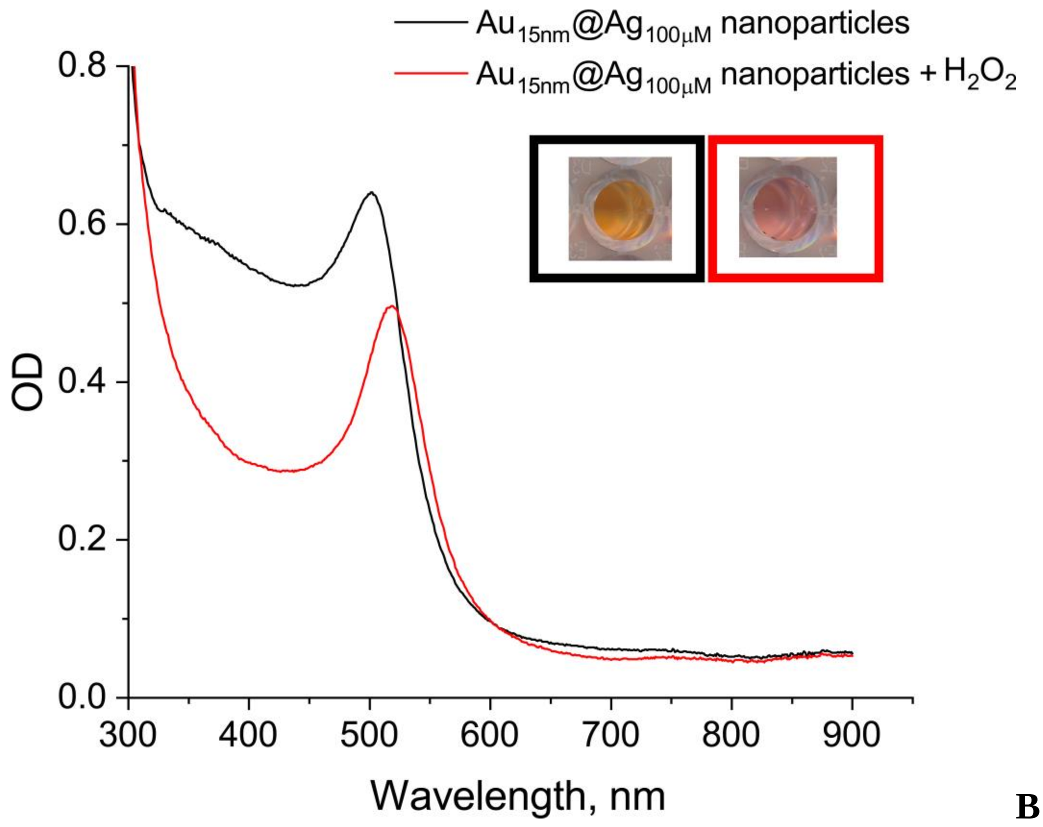

B

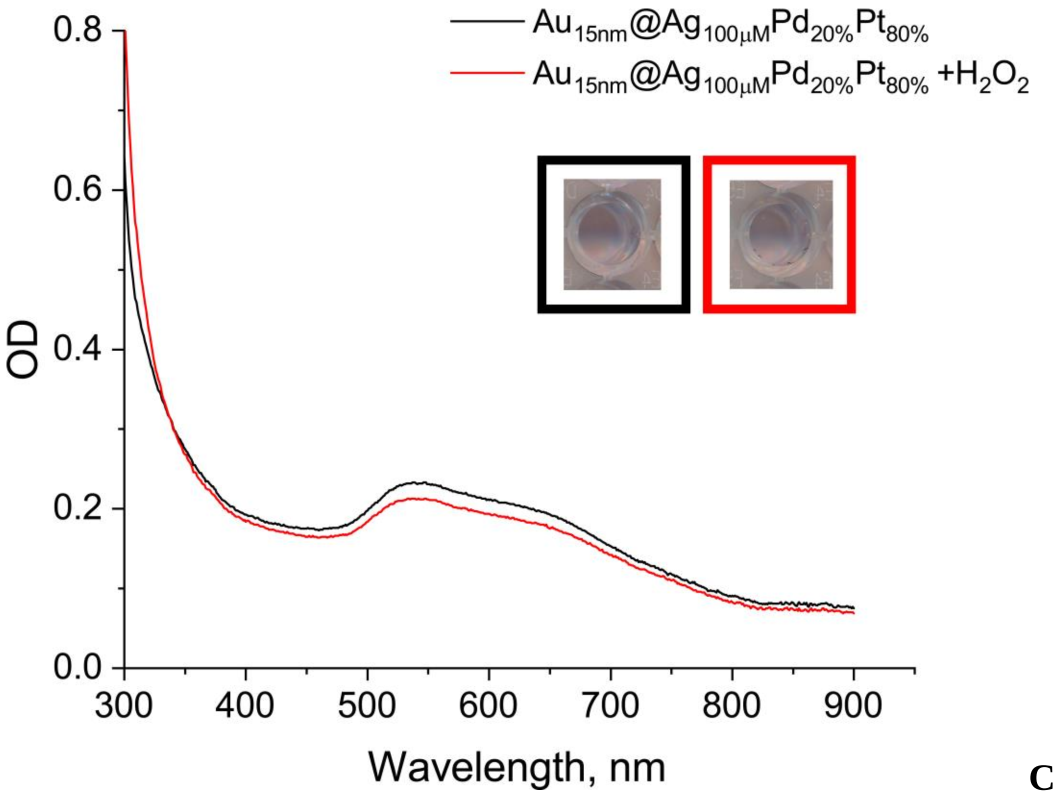

C

Figure S8. Stability assessment via optical spectroscopy. UV-Vis-NIR spectra were recorded for the nanozymes before and after incubation with 100 µM H_2O_2. (A) Au_{15nm} nanoparticles. (B) $Au_{15nm}Ag_{100µM}$ nanoparticles. (C) $Au_{15nm}Ag_{100µM}Pd_{20\%}Pt_{80\%}$ nanoparticles. The corresponding photographs of the colloidal solutions are shown in the insets.

Reproducibility of nanozyme synthesis

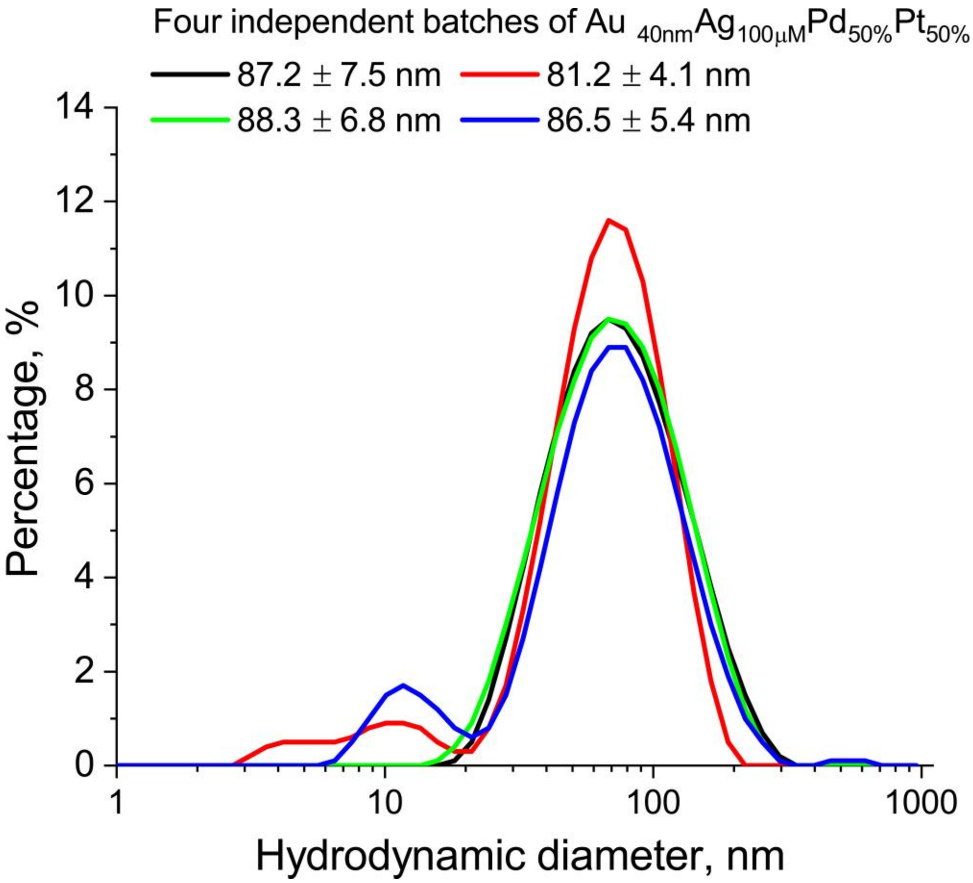

Figure S9. Reproducibility of nanozyme synthesis. Hydrodynamic diameter distributions of four independent batches of $Au_{40nm}Ag_{100\mu M}Pd_{50\%}Pt_{50\%}$ nanozymes measured by dynamic light scattering. The mean diameter for each batch is indicated.

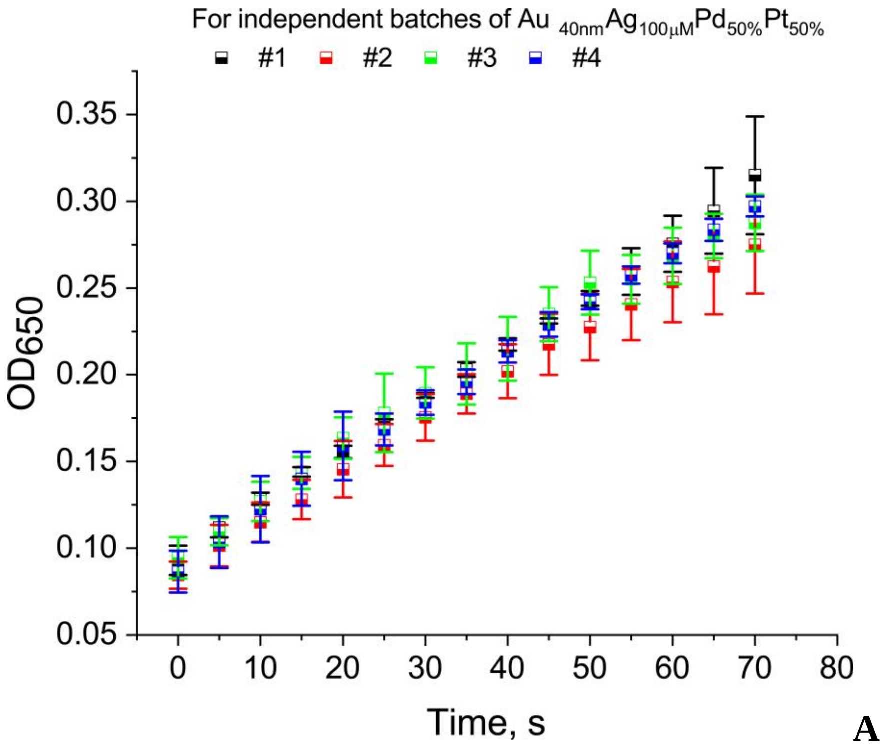

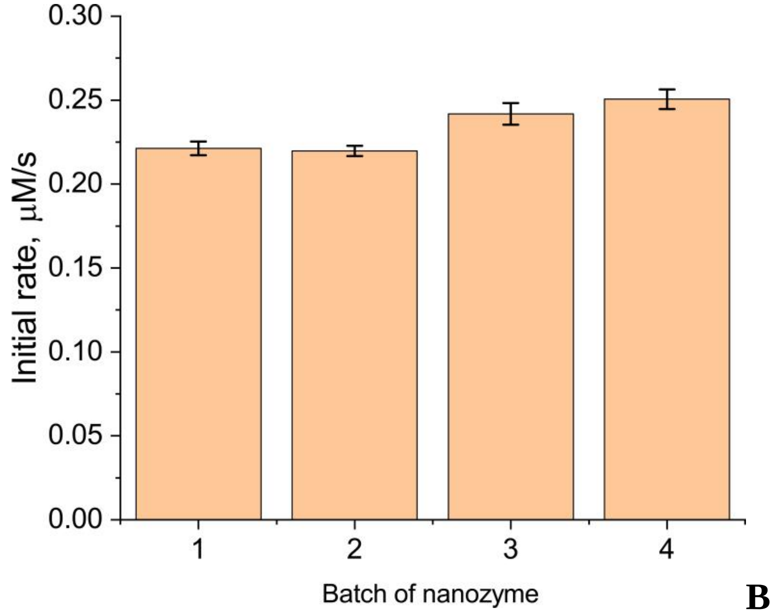

Figure S10. Reproducibility of nanozyme peroxidase-like activity. (A) TMB oxidation curves for four independent batches of Au $_{40nm}$Ag$_{100μM}$Pd$_{50\%}$Pt$_{50\%}$ nanozymes. (B) Corresponding initial reaction rates for the four batches.

References

[1] V.G. Panferov, X. Zhang, K. Wong, J.H. Lee, J. Liu, Biomedical Applications of Nanozymes: An Enzymology Perspective, Angew. Chemie Int. Ed. (2025) e202512409. https://doi.org/10.1002/anie.202512409.

[2] V.G. Panferov, W. Zhang, N. D'Abruzzo, S. Wang, J. Liu, Kinetic Profiling of Oxidoreductase-Mimicking Nanozymes: Impact of Multiple Activities, Chemical Transformations, and Colloidal Stability, ACS Nano 18 (2024) 34870–34883. https://doi.org/10.1021/acsnano.4c12539.

[3] B. Sun, V. Panferov, X. Guo, J. Xiong, S. Zhang, L. Qin, C. Yin, X. Wang, C. Liu, K. Han, S. Wang, H. Jiang, A novel triple-signal biosensor based on ZrFe-MOF@PtNPs for ultrasensitive aflatoxins detection, Biosens. Bioelectron. 267 (2025) 116797. https://doi.org/10.1016/j.bios.2024.116797.

[4] X. Niu, Q. Shi, W. Zhu, D. Liu, H. Tian, S. Fu, N. Cheng, S. Li, J.N. Smith, D. Du, Y. Lin, Unprecedented peroxidase-mimicking activity of single-atom nanozyme with atomically dispersed Fe–Nx moieties hosted by MOF derived porous carbon, Biosens. Bioelectron. 142 (2019) 111495. https://doi.org/10.1016/j.bios.2019.111495.

[5] V.G. Panferov, I. V. Safenkova, A. V. Zherdev, B.B. Dzantiev, Urchin peroxidase-mimicking Au@Pt nanoparticles as a label in lateral flow immunoassay: impact of nanoparticle composition on detection limit of Clavibacter michiganensis, Microchim. Acta 187 (2020) 268. https://doi.org/10.1007/s00604-020-04253-3.

[6] S. Chen, Z. Yu, Y. Wang, J. Tang, Y. Zeng, X. Liu, D. Tang, Block-Polymer-Restricted Sub-nanometer Pt Nanoclusters Nanozyme-Enhanced Immunoassay for Monitoring of Cardiac Troponin I, Anal. Chem. 95 (2023) 14494–14501. https://doi.org/10.1021/acs.analchem.3c03249.

[7] H. Fan, J. Zheng, J. Xie, J. Liu, X. Gao, X. Yan, K. Fan, L. Gao, Surface Ligand Engineering Ruthenium Nanozyme Superior to Horseradish Peroxidase for Enhanced Immunoassay., Adv. Mater. 36 (2024) e2300387. https://doi.org/10.1002/adma.202300387.

[8] L. Jiao, J. Wu, H. Zhong, Y. Zhang, W. Xu, Y. Wu, Y. Chen, H. Yan, Q. Zhang, W. Gu, L. Gu, S.P. Beckman, L. Huang, C. Zhu, Densely Isolated FeN4Sites for Peroxidase Mimicking, ACS Catal. 10 (2020) 6422–6429. https://doi.org/10.1021/acscatal.0c01647.

[9] C. Zhao, C. Xiong, X. Liu, M. Qiao, Z. Li, T. Yuan, J. Wang, Y. Qu, X. Wang, F. Zhou, Q. Xu, S. Wang, M. Chen, W. Wang, Y. Li, T. Yao, Y. Wu, Y. Li, Unraveling the enzyme-like activity of heterogeneous single atom catalyst, Chem. Commun. 55 (2019) 2285–2288. https://doi.org/10.1039/C9CC00199A.

[10] J. Hong, Z. Guo, D. Duan, Y. Zhang, X. Chen, Y. Li, Z. Tu, L. Feng, L. Chen, X. Yan, L. Gao, M. Liang, D. Duan, Highly sensitive nanozyme strip: an effective tool for forensic material evidence identification, Nano Res. (2023). https://doi.org/10.1007/s12274-023-6012-4.

[11] Y. Huang, Z. Ding, Y. Li, F. Xi, J. Liu, Magnetic Nanozyme Based on Loading Nitrogen-Doped Carbon Dots on Mesoporous Fe3O4 Nanoparticles for the Colorimetric Detection of Glucose, Molecules 28 (2023) 4573. https://doi.org/10.3390/molecules28124573.

[12] D. Liu, C. Ju, C. Han, R. Shi, X. Chen, D. Duan, J. Yan, X. Yan, Nanozyme chemiluminescence paper test for rapid and sensitive detection of SARS-CoV-2 antigen, Biosens. Bioelectron. 173 (2021) 112817. https://doi.org/10.1016/j.bios.2020.112817.

[13] X. Meng, S. Zou, D. Li, J. He, L. Fang, H. Wang, X. Yan, D. Duan, L. Gao, Nanozyme-strip for rapid and ultrasensitive nucleic acid detection of SARS-CoV-2, Biosens. Bioelectron. 217 (2022) 114739. https://doi.org/10.1016/j.bios.2022.114739.

[14] Y. Wang, Q. Feng, M. Liu, L. Xue, G. Wang, S. Zhang, W. Hu, N, P, S Codoped Carbon Nanozymes with Enhanced Peroxidase-like Activity and Binding Affinity for Total Antioxidant Capacity Assay, ACS Appl. Nano Mater. 6 (2023) 23303–23312. https://doi.org/10.1021/acsanm.3c04650.

[15] H. Dong, W. Du, J. Dong, R. Che, F. Kong, W. Cheng, M. Ma, N. Gu, Y. Zhang, Depletable peroxidase-like activity of Fe3O4 nanozymes accompanied with separate migration of electrons and iron ions, Nat. Commun. 13 (2022) 5365. https://doi.org/10.1038/s41467-022-33098-y.

[16] L. Jiao, W. Xu, Y. Zhang, Y. Wu, W. Gu, X. Ge, B. Chen, C. Zhu, S. Guo, Boron-doped Fe-N-C single-atom nanozymes specifically boost peroxidase-like activity, Nano Today 35 (2020) 100971. https://doi.org/10.1016/j.nantod.2020.100971.

[17] P. Khramtsov, M. Kropaneva, A. Minin, M. Bochkova, V. Timganova, A. Maximov, A. Puzik, S. Zamorina, M. Rayev, Prussian Blue Nanozymes with Enhanced Catalytic Activity: Size Tuning and Application in ELISA-like

Immunoassay, Nanomaterials 12 (2022) 1630. https://doi.org/10.3390/nano12101630.

[18] Y. Luo, H. Luo, S. Zou, J. Jiang, D. Duan, L. Chen, L. Gao, An In Situ Study on Nanozyme Performance to Optimize Nanozyme-Strip for Aβ Detection, Sensors 23 (2023) 3414. https://doi.org/10.3390/s23073414.

[19] Z. Tao, L. Wei, S. Wu, N. Duan, X. Li, Z. Wang, A colorimetric aptamer-based method for detection of cadmium using the enhanced peroxidase-like activity of Au–MoS2 nanocomposites, Anal. Biochem. 608 (2020) 113844. https://doi.org/10.1016/j.ab.2020.113844.

[20] S.-S. Li, F. Zhao, H. Yu, Z. Xu, Z. Ali, W.-C. Li, Y. Ying, L. Qiao, J. Zheng, J. Li, S.-L. Che, J. Yu, Regulating peroxidase-mimic activity of iron oxide nanozymes through size modulation: electronic structure and specific surface area, Rare Met. (2025). https://doi.org/10.1007/s12598-025-03349-0.

[21] J. Zhang, B. Sun, M. Zhang, Y. Su, W. Xu, Y. Sun, H. Jiang, N. Zhou, J. Shen, F. Wu, Modulating the local coordination environment of cobalt single-atomic nanozymes for enhanced catalytic therapy against bacteria, Acta Biomater. 164 (2023) 563–576. https://doi.org/10.1016/j.actbio.2023.03.040.

[22] Z. Jia, X. Lv, Y. Hou, K. Wang, F. Ren, D. Xu, Q. Wang, K. Fan, C. Xie, X. Lu, Mussel-inspired nanozyme catalyzed conductive and self-setting hydrogel for adhesive and antibacterial bioelectronics, Bioact. Mater. 6 (2021) 2676–2687. https://doi.org/10.1016/j.bioactmat.2021.01.033.

[23] J. Ming, T. Zhu, J. Li, Z. Ye, C. Shi, Z. Guo, J. Wang, X. Chen, N. Zheng, A Novel Cascade Nanoreactor Integrating Two-Dimensional Pd-Ru Nanozyme, Uricase and Red Blood Cell Membrane for Highly Efficient Hyperuricemia Treatment, Small 17 (2021) 1–13. https://doi.org/10.1002/smll.202103645.

[24] J. Xi, R. Zhang, L. Wang, W. Xu, Q. Liang, J. Li, J. Jiang, Y. Yang, X. Yan, K. Fan, L. Gao, A Nanozyme-Based Artificial Peroxisome Ameliorates Hyperuricemia and Ischemic Stroke, Adv. Funct. Mater. 31 (2021) 1–13. https://doi.org/10.1002/adfm.202007130.

[25] Z. Ye, Y. Fan, T. Zhu, D. Cao, X. Hu, S. Xiang, J. Li, Z. Guo, X. Chen, K. Tan, N. Zheng, Preparation of Two-Dimensional Pd@Ir Nanosheets and Application in Bacterial Infection Treatment by the Generation of Reactive Oxygen Species, ACS Appl. Mater. Interfaces 14 (2022) 23194–23205. https://doi.org/10.1021/acsami.2c03952.

[26] S.-B. He, M.-T. Lin, L. Yang, H.A.A. Noreldeen, H.-P. Peng, H.-H. Deng, W. Chen, Protein-Assisted Osmium Nanoclusters with Intrinsic Peroxidase-like Activity and Extrinsic Antifouling Behavior, ACS Appl. Mater. Interfaces 13 (2021) 44541–44548. https://doi.org/10.1021/acsami.1c11907.

[27] B. Jiang, D. Duan, L. Gao, M. Zhou, K. Fan, Y. Tang, J. Xi, Y. Bi, Z. Tong, G.F. Gao, N. Xie, A. Tang, G. Nie, M. Liang, X. Yan, Standardized assays for determining the catalytic activity and kinetics of peroxidase-like nanozymes, Nat. Protoc. 13 (2018) 1506–1520. https://doi.org/10.1038/s41596-018-0001-1.

[28] J. Hao, C. Zhang, C. Feng, Q. Wang, Z.-Y. Liu, Y. Li, J. Mu, E.-C. Yang, Y. Wang, An ultra-highly active nanozyme of Fe,N co-doped ultrathin hollow carbon framework for antibacterial application, Chinese Chem. Lett. 34 (2023) 107650. https://doi.org/10.1016/j.cclet.2022.06.073.

[29] W. Wang, Y. Zhu, X. Zhu, Y. Zhao, Z. Xue, C. Xiong, Z. Wang, Y. Qu, J. Cheng, M. Chen, M. Liu, F. Zhou, H. Zhang, Z. Jiang, Y. Hu, H. Zhou, H. Wang, Y. Li, Y. Liu, Y. Wu, Biocompatible Ruthenium Single-Atom Catalyst for Cascade Enzyme-Mimicking Therapy, ACS Appl. Mater. Interfaces 13 (2021) 45269–45278. https://doi.org/10.1021/acsami.1c12706.

[30] L. Jiao, Y. Kang, Y. Chen, N. Wu, Y. Wu, W. Xu, X. Wei, H. Wang, W. Gu, L. Zheng, W. Song, C. Zhu, Unsymmetrically coordinated single Fe-N3S1 sites mimic the function of peroxidase, Nano Today 40 (2021) 101261. https://doi.org/10.1016/j.nantod.2021.101261.

[31] Y. Chen, L. Jiao, H. Yan, W. Xu, Y. Wu, L. Zheng, W. Gu, C. Zhu, Fe–N–C Single-Atom Catalyst Coupling with Pt Clusters Boosts Peroxidase-like Activity for Cascade-Amplified Colorimetric Immunoassay, Anal. Chem. 93 (2021) 12353–12359. https://doi.org/10.1021/acs.analchem.1c02115.

[32] S. Ji, B. Jiang, H. Hao, Y. Chen, J. Dong, Y. Mao, Z. Zhang, R. Gao, W. Chen, R. Zhang, Q. Liang, H. Li, S. Liu, Y. Wang, Q. Zhang, L. Gu, D. Duan, M. Liang, D. Wang, X. Yan, Y. Li, Matching the kinetics of natural enzymes with a single-atom iron nanozyme, Nat. Catal. 4 (2021) 407–417. https://doi.org/10.1038/s41929-021-00609-x.

[33] X. Zhou, C. Fan, Q. Tian, C. Han, Z. Yin, Z. Dong, S. Bi, Trimetallic AuPtCo Nanopolyhedrons with Peroxidase- and Catalase-Like Catalytic Activity for Glow-Type Chemiluminescence Bioanalysis, Anal. Chem. 94 (2022) 847–855. https://doi.org/10.1021/acs.analchem.1c03572.

[34] L. Huang, Y. Zhou, Y. Zhu, H. Su, S. Yang, L. Feng, L. Zhao, S. Liu, K.

Qian, Dual-modal nanoplatform integrated with smartphone for hierarchical diabetic detection, Biosens. Bioelectron. 210 (2022) 114254. https://doi.org/10.1016/j.bios.2022.114254.

[35] Y. Tang, Y. Wu, W. Xu, L. Jiao, Y. Chen, M. Sha, H.R. Ye, W. Gu, C. Zhu, Ultrathin Ruthenium Nanosheets with Crystallinity-Modulated Peroxidase-like Activity for Protein Discrimination, Anal. Chem. 94 (2022) 1022–1028. https://doi.org/10.1021/acs.analchem.1c03987.

[36] F. Feng, Y. Zhang, X. Zhang, B. Mu, J. Zhang, W. Qu, W. Tong, M. Liang, Q. An, Z. Guo, L. Zhao, Enhancing the peroxidase-like activity of MoS2-based nanozymes by introducing attapulgite for antibacterial application and sensitive detection of glutathione, Nano Res. 17 (2024) 7415–7426. https://doi.org/10.1007/s12274-024-6685-3.
